# Honeybees’ foraging choices for nectar and pollen revealed by DNA metabarcoding

**DOI:** 10.1038/s41598-023-42102-4

**Published:** 2023-09-07

**Authors:** Matti Leponiemi, Dalial Freitak, Miguel Moreno-Torres, Eva-Maria Pferschy-Wenzig, Antoine Becker-Scarpitta, Mikko Tiusanen, Eero J. Vesterinen, Helena Wirta

**Affiliations:** 1 Institute of Biology, Karl-Franzen University of Graz, Universitätsplatz 2, 8010 Graz, Austria; 2 Institute of Environmental Systems Science, Karl-Franzens-Universität Graz, Merangasse 18/I, 8010 Graz, Austria; 3 Institute of Pharmaceutical Sciences, Pharmacognosy, University of Graz, Beethovenstraße 8, Graz, Austria; 4 CIRAD, UMR PVBMT, 97410 Saint Pierre, La Réunion France; 5 Department of Evolutionary Biology and Environmental Studies, University of Zurich, Zürich, Switzerland; 6 Department of Biology, University of Turku, Vesilinnantie 5, Turku, Finland; 7 Department of Agricultural Sciences, University of Helsinki, Latokartanonkaari 5, P.O. Box 27, 00014 Helsinki, Finland

**Keywords:** Ecology, Agroecology, Molecular ecology

## Abstract

Honeybees are the most widespread managed pollinators of our food crops, and a crucial part of their well-being is a suitable diet. Yet, we do not know how they choose flowers to collect nectar or pollen from. Here we studied forty-three honeybee colonies in six apiaries over a summer, identifying the floral origins of honey and hive-stored pollen samples by DNA-metabarcoding. We recorded the available flowering plants and analyzed the specialized metabolites in honey. Overall, we find that honeybees use mostly the same plants for both nectar and pollen, yet per colony less than half of the plant genera are used for both nectar and pollen at a time. Across samples, on average fewer plant genera were used for pollen, but the composition was more variable among samples, suggesting higher selectivity for pollen sources. Of the available flowering plants, honeybees used only a fraction for either nectar or pollen foraging. The time of summer guided the plant choices the most, and the location impacted both the plants selected and the specialized metabolite composition in honey. Thus, honeybees are selective for both nectar and pollen, implicating a need of a wide variety of floral resources to choose an optimal diet from.

## Introduction

Most of the wild and cultivated flowering plant species depend on animal pollinators^1, 2^. Honeybees are the most abundant pollinators in the world^2^ and are kept by humans for their production of honey as well as for pollination they provide. Because of their high abundancy in a variety of environments, honeybees are important pollinators for crop plants^3^. At the same time the availability of proper nutrition contributes to the health of honeybees. A variety of food sources is beneficial for bee health^4^, yet in modern agricultural environments monocultures are common, which might compromise proper nutrition for the bees^5^. Furthermore, it has been shown that bees stressed by pesticides prefer more variable food^6^, which suggests that a diverse nutrition is not only important for normal functioning, but could also promote bee health in times of stress.

Honeybees collect nectar and pollen to fill different nutritional needs, those of carbohydrates and those of proteins and lipids. Nectar mostly consists of monosaccharide sugars, namely glucose and fructose. Nectar is used to support the energetic needs of the colony, such as the costly flight of the foragers and thermoregulation of the hive^7^. Honeybees commonly select plants for foraging nectar based on the sugar concentration of the nectar^8^ and the total sugar content within and between plant species can vary extremely, from 6.3 to 85%^9^. The amount of proteins and lipids as well as the composition of different amino and fatty acids also vary greatly between pollen from different plant species. Protein content in bee collected pollen varies from 1.5 to 48.4% and lipid content from 1.2 to 24.6%^10^. The pollen preferences of the foragers are determined by the requirements of the colony; preferred pollen sources are influenced more by the composition of fatty and amino acids of the pollen than by the total protein content^11–13^. Like nectar, foraged pollen is stored in the hive, but it is mainly used in feeding the developing brood, while adults may survive longer without pollen^7^.

Not only are nectar and pollen used for different purposes, but their foraging is also performed by different sets of individuals, as individual forager bees typically only forage either nectar or pollen^14, 15^. Foraging nectar and pollen are thus separate processes, also from the perspective of the plants that produce them. As nectar and pollen differ in terms of nutrients they contain, the nectar and pollen reward plants offer may be very different across species. Some plants, such as wind pollinated species, do not produce nectar at all, but may still act as a source for pollen^16^. The plant sources may then differ in quantity and quality between the offered nectar and pollen reward. When also considering the fact that these two resources are used for different purposes and are foraged by different individuals, it would be expected that different plants are used for nectar and pollen foraging. Yet most research examines the selection of one resource type at a time (e.g., pollen^17^ or nectar^18^). However, some studies have looked how bees select plants for nectar and pollen, yet with either a very restricted number of colonies^19^ or at single time point, in the spring^19, 20^. As the availability of plants changes during the summer due to different flowering times, changes in foraging are also likely to happen throughout the season^19^. Thus, there is a lack of thorough understanding of whether honeybees select different plants for the two types of resources, and how selective they are for each resource type from the available flowers, as honeybees are known to utilize only a part of the available resources^21^.

Previous studies on foraging choices of honeybees have been based on morphological identification of pollen grains in honey (melissopalynology)^22^. Pollen from the nectar source plant may attach to the forager bee and later end up in the honeycomb. Today, DNA-based methods allow extraction and more precise taxonomic identification of the plant origin of the honey, not only from pollen but from any plant tissue^17^. DNA can also be readily extracted from hive-stored pollen, beebread^23^. Bees prepare beebread from pollen by mixing it with glandular secretions and small amounts of nectar. Although some natural cross-contamination of these resources is inevitable, we can use the DNA in honey to infer the source plants of nectar, while the DNA in beebread can be used to infer the source plants of pollen^17, 22–25^.

In addition to the nutrients in nectar and pollen, plants produce a wide variety of specialized metabolites that also end up in plant provided resources, like nectar. Some specialized metabolites may affect pollinator behavior and improve pollination success of the flowering species^26^, but some of them also act as deterrents of pollinators, creating a paradoxical situation, as plants also need to attract pollinators^27^. It is yet somewhat unclear why deterrent compounds end up in nectar, although they could potentially protect the nectar from unwanted visitors or control microbial activity^28^. Overall, the role of plant specialized metabolites in honeybee foraging choices and their effects on honeybee colonies is unresolved^29^.

Here we use DNA metabarcoding to identify the plant origin of both honey and beebread storages of honeybee colonies to examine the foraging choices for these two resources simultaneously, at different times of the summer and in different locations. By comparing the plants found to be used to the surrounding flower availability, we determine how selective honeybees are when foraging for nectar and for pollen. We also examine what types of specialized metabolites are found in the honey samples using ultra-high-performance liquid chromatography-high resolution mass spectrometry (UHPLC-HRMS). Further, we examine how the foraging choices change across the flowering season for both nectar and pollen and assess how time and location affect the foraging choices as well as the specialized metabolite composition in the honey.

## Results

### Summary of methods

We collected honey and beebread samples from 43 beehives, located in six apiaries within three areas at the beginning of June, July, and August in 2021. From the time of foraging to our sampling, we assume a similar amount of time for both sample types, as the turnover rate for beebread is about 2 weeks during the summer months^30^, and as it takes 3–10 days for bees to process nectar into honey^31^. The three areas were located in southwestern Finland, approximately ten kilometers apart from each other, thus further apart than bees typically fly for foraging. To identify the plant taxa in the samples we used DNA metabarcoding based on the gene *internal transcribed spacer 2* (ITS2). We also mapped the natural flowering plants around the three apiary areas at the same time points as the sampling, to compare the plants available to the ones detected in the samples. We collected additional honey samples in August to examine the specialized metabolites in the honey with UHPLC-HRMS, using two purification methods to achieve wide coverage of compounds.

The analyses were conducted at the taxonomic level of genus using relative read abundances of genera^32^ and presence-absence data to ensure the robustness of the results and interpretation, as the DNA metabarcoding method can generate some biases in relative read abundances among taxa^33^. Beebread and honey composition data were graphically compared using nonmetric multidimensional scaling (NMDS), and the multivariate homogeneity of group dispersions was tested using the PERMDISP procedure. Then, we tested the change in plant composition between the sample types using a permutational analysis of variance (PERMANOVA) with Hellinger distances. As a complementary honey and beebread composition analysis, we identified indicator species that characterized each sample type using the IndVal procedure. The richness of genera within a hive in the two sample types was compared with linear mixed models and the number of shared genera with binomial generalized linear mixed model. To assess the proportion of available flowering plants used for nectar and pollen foraging, we compared flowering plants mapped in the surroundings to the ones detected in the samples using euler-diagrams. To find the factors affecting the foraging choices we again applied NMDS’s and used redundancy analysis (RDA) to assess the influential variables, with Hellinger-transformed values. To find whether apiary area also has an effect on the composition of specialized metabolites in honey, we used multivariate data analysis. The most abundant specialized metabolites discriminating the three apiary areas as well as the most abundant ones commonly occurring in all three apiary areas were annotated by comparing their mass spectrometry (MS) data with literature and databases.

### Taxa detected in honey and beebread

After bioinformatic filtering, there were 2,592,723 sequencing reads from the honey samples and 1,724,302 from the beebread samples. After filtering out samples with fewer than 5,000 reads (n = 5), we had 97 honey samples for analyses, with on average 26,656 (SD ± 10,877) reads per sample. Of these 39 were collected in June, 30 in July and 28 in August. For beebread we had 87 samples after the filtering with on average 19,769 (SD ± 7,249) reads per sample. 36, 28 and 23 of these were collected in June, July and August, respectively. 95.1% of honey and 91.1% of beebread reads were assigned to a genus. As the proportions assigned to species were far lower (14.9% for honey and 21.8% for beebread), in order to use most of the data available, we use the genus level assignments for all analyses.

The total number of different genera detected in either honey or beebread was 67 (57 in honey, 61 in beebread). In honey samples we found 33, 33 and 42 genera, while in beebread samples we found 39, 20 and 29 genera in June, July and August, respectively (Supplementary Table S1). Over the whole season, almost half of the sequencing reads in honey came from two genera, 32.8% originating from *Brassica* and 17.4% from *Rubus*. In beebread, most reads also originated from two genera, 27.1% from *Brassica* and 22.1% from *Sorbus*. The relative abundances of genera in the samples at each time point can be found in supplementary table S1. In terms of plant diversity specific to the apiary areas, the number of genera found in honey were 17, 25 and 29 in the apiary area A-B in June, July and August, respectively. For apiary area C–D there were 23, 15 and 20 genera, and for E–F 26, 23 and 29 genera, in June, July and August. As for beebread, the number of genera in the area A–B was 24, 15 and 21, for the area C–D 28, 10 and 15, and for the area E–F 28, 10 and 20 genera, in June, July and August, respectively.

### Shared and distinct plant genera found in honey and beebread

**Figure 1:**
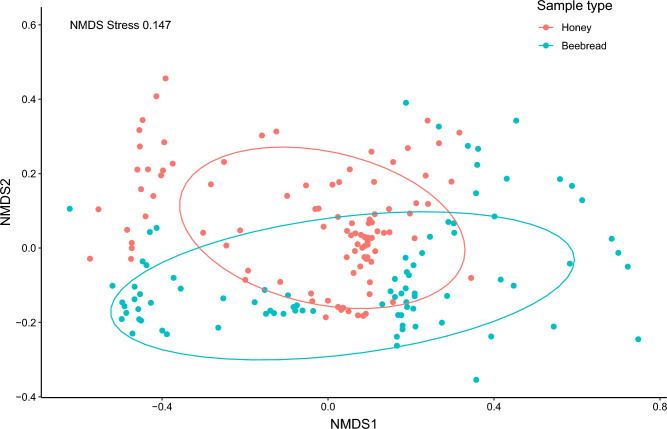
Plant genera composition of honey (red, n = 97) and beebread (blue, n = 87) samples collected from 43 hives in 6 apiaries in Finland in 2021. Figure showing non-metric multidimensional scaling (NMDS) based on Hellinger dissimilarity index. Ellipses show 75% confidence limits for each sample type.

Out of the 67 total genera, 51 were detected in both honey and beebread (Supplementary Table S1). Six genera were found only in honey and ten only in beebread. Regardless of the partial overlap in plant genera composition for both honey and beebread, as shown by the ordination (Fig. 1), the plant communities in honey and beebread samples differ significantly in their dispersion (PERMDISP, F = 12.575, *p* < 0.001, honey = 0.726, beebread = 0.811). We found a significant difference in plant genera composition between honey and beebread (PERMANOVA, F = 24.961, *p* = 0.001, R^2^ = 0.070), although this result is at least partly due to the difference in group dispersions shown by the multivariate homogeneity of group dispersion analysis (Supplementary Fig. S1).

**Figure 2:**
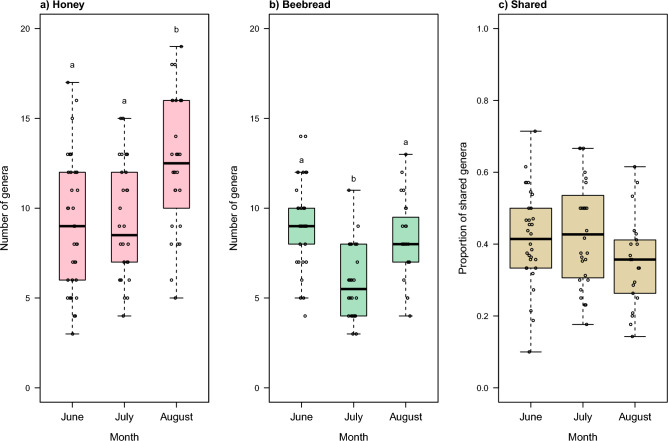
The number of genera in samples collected from beehives in Finland in 2021 at each time point from 6 apiaries, showing genera in honey (**a**, June n = 39, July n = 30, August n = 28) and beebread (**b**, June n = 36, July n = 28, August n = 23), and the proportion of shared genera for paired samples of honey and beebread from individual hives (**c**, June n = 32, July n = 28, August n = 21). Significantly different groups in pairwise comparisons in (**a**) and (**b**) are denoted with letters.

To assess how the plant choices change through time in colonies, we analyzed the number of genera in samples of honey and beebread from individual hives. Honey samples had a larger number of genera in August than at earlier time points (Fig. 2A, Supplementary Table S2) while beebread samples had the lowest number of genera in July in comparison to the other time points (Fig. 2B, Supplementary Table S3). Overall, the number of genera was significantly higher in honey samples (mean 10.16) than beebread samples (mean 7.87) (Supplementary table S4). To assess the proportion of plant genera shared between the honey and beebread communities in each hive, we analyzed paired samples of honey and beebread, collected from individual hives at the same time (June n = 32, July n = 28, August n = 21). The proportion in hives ranged from 0.10 to 0.71 but was on average similar throughout the summer, ranging from 0.35 to 0.43 between time points (Fig. 2C). There was no significant difference in the proportion of shared genera among the time points (GLMM, *p* = 0.12, Supplementary Table S5).

Based on the indicative species analyses, to identify which plant genera are mostly selected either for nectar or pollen foraging, twelve genera are significantly associated with honey samples, compared to eight genera for beebread (Table 1, for results based on presence-absence data see Supplementary Table S6). The genera most strongly associated with honey were *Rubus* and *Myosotis*, whereas for beebread they were *x-Amelasorbus* (a hybrid genus) and *Pisum*.

**Table 1:** Statistically supported indicative plant genera associated with either honey (n = 97) and beebread (n = 87) samples that were collected from 43 hives in 6 apiaries in Finland in 2021, based on relative read abundances, with component values.

Honey	A (specificity)	B (fidelity)	stat	*p* value
Rubus	0.936	0.845	0.889	0.001
Myosotis	0.971	0.474	0.678	0.001
Salix	0.647	0.505	0.572	0.016
Vicia	0.628	0.485	0.551	0.004
Taraxacum	0.888	0.268	0.488	0.001
Prunus	0.754	0.309	0.483	0.002
Malus	0.654	0.289	0.434	0.031
Chamaenerion	0.796	0.165	0.362	0.014
Rosa	1.000	0.124	0.352	0.001
Medicago	0.925	0.082	0.276	0.016
Populus	1.000	0.062	0.249	0.028
Comarum	1.000	0.062	0.249	0.031

Beebread	A (specificity)	B (fidelity)	stat	*p* value
x-Amelasorbus	0.757	0.379	0.536	0.001
Pisum	0.724	0.391	0.532	0.026
Calluna	0.856	0.322	0.525	0.001
Rhododendron	0.690	0.253	0.418	0.024
Syringa	0.808	0.161	0.361	0.009
Cirsium	0.771	0.161	0.352	0.035
Crataegus	1.000	0.092	0.303	0.002
Aronia	1.000	0.069	0.263	0.010

### Selectivity of floral choices from the available flowers

**Figure 3:**
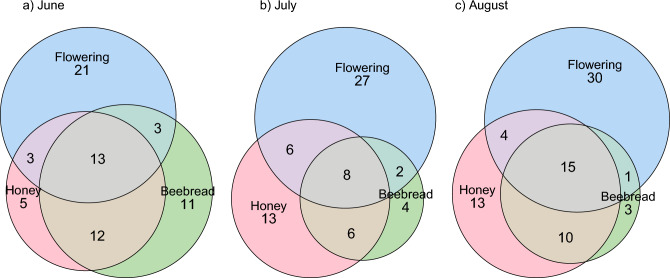
Number of shared and unique plant genera found in samples of honey (June n = 39, July n = 30, August n = 28) and beebread (June n = 36, July n = 28, August n = 23), collected in 43 beehives in six apiaries in Finland in 2021, and the number of flowering plants surrounding the hives. Honey shown in pink, beebread in green and flowering plants in blue at different times; (**a**) June, (**b**) July and (**c**) August.

During the surveys of flowering plants in the 28 plots presenting the six different habitat types surrounding the apiary areas, we found 99 species, representing 73 genera and 27 families (Supplementary Table S7). 39 genera were in flower in June, 43 in July and 50 in August. The agricultural fields found flowering close to the hives were *Linum usitatissimum*, *Brassica* sp. and *Solanum tuberosum*, all in July. Out of the 73 genera found, less than half were found in the honey (32) or beebread (30) samples. The proportions of flowering plant genera that were also found in honey was 40.0%, 32.6% and 38.0% in June, July and August (Fig. 3). In beebread the proportion of flowering plants was 40.0%, 23.3% and 32.0% in June, July and August. Out of the genera found in honey and beebread, 33 genera were not found during the flowering plant survey. The proportion of genera not found flowering in the natural habitat types but found in honey samples in June, July, and August, were 51.5%, 57.6% and 54.8% and in beebread samples 59%, 50% and 44.8%. On the other hand, most of the plants found flowering in the natural habitats were not found in either honey or beebread, being 52.5%, 62.8% and 60.0% in June, July and August, respectively (Fig. 3).

### Impact of the time of the season and location on the floral choices

**Figure 4:**
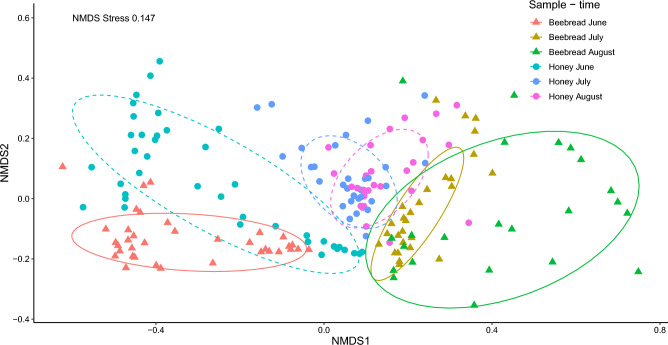
Plant genera composition of beebread (triangles, dashed ellipses, June n = 36, July n = 28, August n = 23) and honey (circles, solid ellipses, June n = 39, July n = 30, August n = 28) at different time points in samples collected from 43 hives in 6 apiaries in Finland in 2021. Figure showing non-metric multidimensional scaling (NMDS) based on Hellinger dissimilarity index, ellipses showing 75% confidence limits.

The selection of flowers by honeybees strongly varies between sample type and change over time (Table 2). In the variation partitioning, sample type and time explained 30.7% of the total variation, while the variables associated with the experimental design and spatial effects (i.e., site, apiary and hive) accounted for 1.2% of the total variation, and 62.5% are not explained by the model (RDA model F = 25.31, df = 5, *p*-value <  = 0.001, adj R^2^ = 45.7, Table 2, Fig. 4).

**Table 2:** Results from the partial canonical model including spatial variables to control pseudoreplication linked to experimental design (see statistical methods). Variation partitioning quantifies the proportion of variation explained by sample type, sampling time and variables (site, apriary, hive) on the plant genera found in samples of honey (n = 97) and beebread (n = 87), collected from 43 hives in 6 apiaries in Finland in 2021.

Variables	Df	Variance	F	*p*-value
sample_type	1	0.043	25.535	**0.001**
time	2	0.145	42.636	**0.001**
sample_type:time	2	0.052	15.204	**0.001**
Residual	135	0.229		

Fractions	Df	Adj. R^2^		
X1 = Sample_type & Time	3	0.307		
X2 = Area, apiary & hive	42	0.012		
X1 + X2	45	0.375		
Residuals		0.625		

Significance values are [bold].

### Composition of specialized metabolites in honey

The most abundant specialized metabolites occurring commonly across all apiary areas in the honey samples collected in August were annotated based on the MS data (Supplementary Text S1, Table S10). Multiple isomeric monoterpene glycosides and tricoumaroyl spermidines, carboxylic and dicarboxylic acids, the plant hormone abscisic acid and the vitamin pantothenic acid were among the major metabolites found in all three apiary areas (Supplementary Table S9, Figs. S6 and S7).

**Figure 5:**
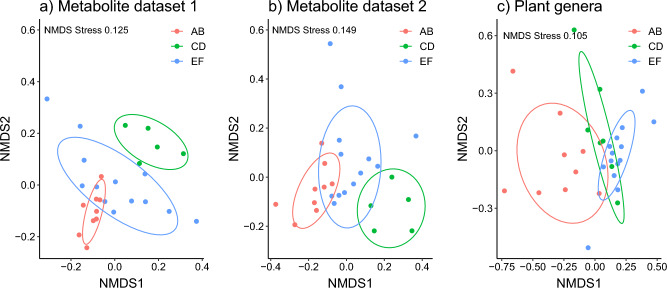
Non-metric multidimensional scaling (NMDS) plots for specialized metabolites in honey with two separation techniques producing dataset 1 (**a**, n = 27), dataset 2 (**b**, n = 27) and plant genera composition in honey samples at the same time point (**c**, n = 30), grouped by apiary sites. Ellipses show 75% confidence limits for each site.

The impact of the apiary area on the specialized metabolite composition in honey was assessed by multivariate data analysis (Supplementary Text S1, Figs. S4, S5, S8 and S9), showing not only the occurrence but also the abundance of specific metabolites to differ between the apiary areas. The most abundant discriminant metabolites were deduced from OPLS-DA models using the apiary areas as classifiers, and annotated (Table S11, S12). Their distribution among the three apiary areas was found to vary strongly (Supplementary Figs. S8 and S9). Using NMDS the specialized metabolites from both datasets seem to cluster in similar fashion, while the plant genera in honey samples from the same time points overlap more in apiary sites CD and EF (Fig. 5).

## Discussion

Nectar and pollen constitute the whole diet of honeybees, yet the nutritional contents, as well as quantities of nectar and pollen differ greatly among plant species. Thus, the choices honeybees make, when selecting flowers to collect nectar or pollen from, are very important for their diet. Here we found that honeybees largely choose the same plants for both nectar and pollen, when considering all the hives across the whole summer. Yet, when focusing at individual colonies at a time point the plants chosen for nectar or pollen differ substantially. We also show that honeybees use only a fraction of the available flowers in the surrounding natural habitats, for either nectar or pollen foraging. In our study the time of the summer was the largest determinant on which plants were foraged on, but the foraged resource type and location also played a significant role. A large variety of specialized metabolites was found in the honey samples, showing differences among the apiary areas. Below we discuss all these findings in turn.

### Honeybees are more selective for pollen

We found that honeybee colonies use different plants for nectar and for pollen, as on average less than half of the plant genera found in a hive at a time were found in both honey and beebread samples. These differences are not surprising, as nectar and pollen are collected for different needs, by different specialized foragers, and nectar and pollen nutritional qualities as well as quantities vary widely among plants^34^. The average number of genera in honey samples in the colonies was higher than in beebread. However, the composition of genera in beebread samples varied significantly more. Therefore, while fewer genera were used for pollen foraging, the genera varied more between hives and time points, making the overall number of plant genera used for pollen higher. This result corresponds to previous studies finding that honeybees forage on fewer species for nectar than for pollen^19, 20, 35^. This together with our results suggests that same plants are more consistently used for nectar, while the foraging choices of pollen change more frequently. This could mean that pollen foraging sources are more variable to maintain the flow of important nutrients required by the colony, as honeybee colonies can remedy deficiencies in essential fatty and amino acids by preferring pollen that complement the deficiencies^11, 12^. We further detected a number of genera being strongly associated with one resource type, suggesting the properties of nectar or pollen of these plants to be favorable^19, 20, 35^.

Although about half of the genera detected in colonies at a time was different for nectar and pollen, we found that as a whole largely the same plant genera were utilized for both nectar and pollen foraging. This^36^ indicates that many plant genera are suitable for both pollen an nectar foraging, but the resource itself is important, as we see clear differences in the choices for the two resource types.

### Limited use of the available floral resources

Throughout the summer fewer than half of the available flowering natural plants were found to have been foraged on for either nectar or pollen. Previously, DNA based comparisons of what honeybees forage on from the surrounding floral resources have similarly found that only a fraction of the available flowers is used by honeybees^21, 37^. For example, a study in a botanic garden in Wales found that honeybees used only 11% of the available flowering taxa, preferring native or near-native plants^21^. In our study we similarly found that horticultural plants were not majorly used by bees. Only two genera had relative abundance greater than 1% at any point in either honey or beebread, *Hydrangea* (2.2%) and *Phacelia* (2.3%), which is sometimes planted as a resource for honeybees.

On the other hand, we found that honeybees had used many genera that we had not detected in our surveys of flowering plants in natural habitats. Eight of such genera were ornamental or other garden plants, such as *Rosa* and *Paeonia,* and six were cultivated plants, such as *Coriandrum* (coriander) and *Raphanus* (radish), showing that honeybees forage also in gardens and on fields in our study area. This is expected, as also in previous studies in the UK honeybees have been shown to use garden plants extensively^38, 39^. Ten genera found in honey and beebread samples, but not recorded during our survey of flowering understory plants, were typical Finnish trees. Yet, a few native Finnish flowering genera were found commonly in the honey samples, such as *Persicaria* (knotweeds), *Fallopia* (e.g., black bindweed) and *Convallaria* (lily-of-the-valley), although we had not recorded them in the survey. This means our flowering plant surveys were not thorough enough to give a full picture of the available floral resources, even though we assessed all the habitat types in the area. Yet, honeybees only used a fraction of the flowers that we did find flowering, indicating honeybees are selective in choosing plants to forage on, in support of other studies showing honeybees to be selective^21, 37^.

### Choices for nectar and pollen change according to time of the summer differently

The time of the summer was the strongest determining factor of plant genera found in honey and beebread samples. This is an expected result considering the different flowering times of plants, changing the availability during the year, and in line with previous research finding honeybees’ flower usage to clearly follow plant phenologies^38, 40^. Location also played a role in the usage of plants, as the spatial effects accounted for almost one fourth of the variation. This was anticipated since the flowering plant pool within reach of a colony would be defined by the site.

The diversity of plants used for nectar and pollen foraging, detected in honey and beebread samples, had different dynamics through the summer. The number of genera in honey was highest in August, which was also when most flowering plants were detected in the environment, but after the main nectar flow in Finland^41^. However, in beebread samples we found fewer genera in July, when the rapeseed (*Brassica*) flowers. A similar effect in reduced pollen richness coinciding with the mass flowering of rapeseed has been seen in other studies as well^42^. This has been explained by optimal foraging theory, predicting that when a preferred food source is abundantly available, foragers should utilize that, and when the preferred resource becomes limited, the number of utilized species would increase^40^. This is supported by our observations, as in July rapeseed also dominated the read abundance in beebread, and the diversity of genera in beebread was at its lowest. As rapeseed appears to be a good source of nutrition for honeybees^43^ a preference could be expected, yet such preference has often not been shown^18, 35^. Interestingly in honey samples the overall number of genera did not dip in July. It was slightly lower in apiary area E–F, and clearly lower in area C-D, which is surrounded by the lowest amount of agricultural landscape. These differences could result from the different landscape imposing different resource availability between the areas, shaping the breadth of foraged plants^40^. Nevertheless, the result shows the availability of an abundant source affects the choices of pollen foragers differently from nectar foragers.

The lower number of genera in beebread in July may appear alarming, because the diversity of the pollen diet has been linked to honeybee health^44^. However, lower diversity of pollen does not always cause problems, as the nutritional quality of pollen is more important^4^. For example, the mass flowering of maize is detrimental to honeybee health, because maize pollen is of low quality^4^, but a pollen diet of similar low diversity does not cause detrimental health effects when composed of better-quality sources^4^. Diverse sources of pollen during the mass flowering are thus especially important in areas where the hives are close to crops that produce pollen with low nutritional content, and at times of resource limitations^40^.

### Natural DNA contaminations between honey and beebread in the hive

When honeybees forage, pollen from the flower attaches to them, and some of it may also enter the combs as honeybees are processing nectar into honey. Also, honeybees add small amounts of nectar and glandular secretions to pollen as they prepare it as beebread^7^. Thus, the beebread samples could contain traces of DNA from the plants used as nectar sources and vice versa. Such possible natural contaminations will make the plant composition of the two sample types more similar, making differences detected more conservative. We nevertheless find differences in plant genera in honey and beebread samples, both based on relative read abundances and occurrences, and suspect that due to the mentioned biases the actual foraging choice differences are stronger.

### Specialized metabolite composition in honey is influenced by apiary location

Like the plant genus composition, the specialized metabolite profiles of honey samples were influenced by location, suggesting that the availability of the different plant taxa in each apiary area contributed to the observed differences. It was consistent with the perceived similarity of the surrounding landscape, areas A–B and E–F having a similar land use composition. Interestingly, the specialized metabolites appear to cluster more distinctly when compared with the plant genera composition, although it could be explained by the slightly different sampling methods. Many of the annotated compounds have previously been detected in honey samples. For example, numerous hydroxcinnamoylamines, known pollen constituents with varying levels and substitution patterns between plant species^45–47^, were annotated. Some of the detected isomers with obvious floral origins consistently occurred in samples from all three areas (e.g. tricoumaroylspermidine isomers are known to occur in rapeseed beebread^48^), while others obviously were derived from more specifically occurring plant taxa. Commonly occurring were also e.g., pantothenic acid, a vitamin, and abscisic acid, a plant hormone possessing diverse and important regulatory roles in plants. Interestingly, abscisic acid seems to have a beneficial impact on bee health. Abscisic acid supplementation has been shown to enhance the immune response in honeybees and to contribute to colony fitness^49^, and it was able to enhance cold stress tolerance in in-vitro reared honeybee larvae^50^, thus it could have guided the floral choices.

Two carboxylic acids commonly occurring across apiary areas are traced back to royal jelly, the larval feed of the honeybee, which is known to contain decene- and decanedioic acids identified herein^51, 52^. These compounds have been previously detected in various honey accessions, and their occurrence in royal jelly suggests that they originate from the bee itself, and not be playing a role in the foraging choices.

Among the metabolites that discriminate among the areas, the flavonoids chrysin, tectochrysin and pinobanksin have frequently been detected in honey^53, 54^. Since they are known as typical propolis constituents, their abundance in honey may rather depend on its propolis content than on its floral origin^55^. Vomifoliol has previously been detected as major constituent in honey produced from *Salix* nectar^56^ and as minor constituent in *Trifolium pratense* honey^57^. In line, *Salix* and *Trifolium* were among the relatively most abundantly found genera in the DNA of these honey samples. Salicin has only occasionally been detected in honey, but is also known as constituent of *Salix* species^58^.

## Conclusions

We found honeybees to be clearly selective for flowers overall, using only a fraction of the plants available. The mass-flowering of rapeseed seemed to alter pollen foraging more than nectar. The time of the summer and the surroundings of the hives determine flower availability, and although many plant species could provide both suitable nectar and pollen, we found each colony to select largely different plants for nectar and for pollen. These together tell that honeybees base their foraging choices on multiple factors, and that they actively choose the plants they forage on. Further research on honeybee nectar and pollen requirements and foraging choices simultaneously could aid bee conservation efforts. As we know that a diversity of plants is important to fulfill the nutrient needs of honeybees, we need to assure a wide variety of plants are available for them throughout the summer, so that they can select the right plants to fill their resource needs.

## Materials and methods

### Description of habitat and sampling

**Figure 6:**
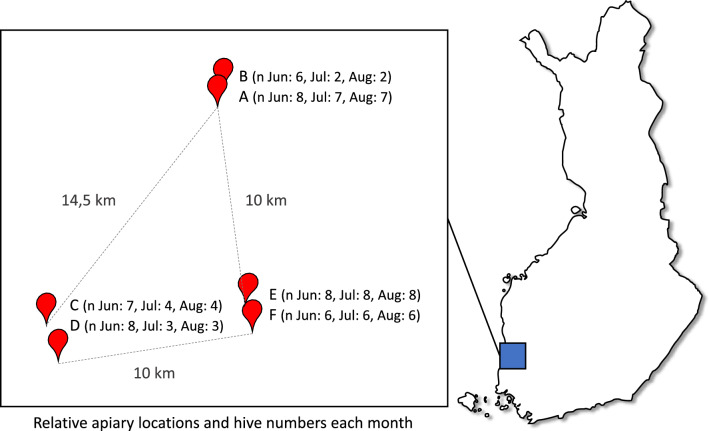
The study area location in Finland (rectangle on map not to scale) with relative locations of the apiaries with hive numbers each month (June, July and August of 2021).

To study the foraging choices of honeybees for nectar and pollen, we studied 43 honeybee colonies and the surrounding flowering plants in Southern Finland in 2021. The hives were established in 2020 and maintained by two experienced beekeepers using conventional Finnish beekeeping practices^41^. The hives were located in six apiaries, with two apiaries less than 2 km apart and 10–15 km between each pair of apiaries (Fig. 6). In each apiary there were two to eight hives. The hives were also used in an experiment on trans-generational immune priming^59^. The priming treatment had no effect on foraging behavior (Supplementary Fig. S2) and is thus not further considered in this study.

As honeybees mainly forage within a few kilometers of their hive^60^ and rarely go beyond ten kilometers^61^, we consider each pair of apiaries to partly share the floral resources, while between the pairs of apiaries the studied honeybees would mostly be too far from each other to use the same flower resources. The apiary area consists of a mosaic of cultivated fields and managed forests, with differences in the ratios of these between the vicinity of each apiary, as determined by Corine land cover database (version 20b2, 2018, European 100 m raster database^62^; Supplementary Fig. S3).

We mapped the flowering plants and from the bee hives we collected beebread and honey samples at three time points during the summer 2021. The first sampling from the hives and flower counts were done in June (8.–13.6.), second in July (9.–14.7.), and third in August (10.–13.8.2021). For the sampling, which was only from the hives, we had the permission of the owners of the hives and thus it complied with all national rules and legislation in Finland, as no other permits are required. The flowering plants were assessed within 3 km distance of the hives, in sites selected by stratified (random) sampling with arbitrary allocation, in different habitat types indicated by Corine land cover database. Selected habitat types to map were mixed forests, conifer forests and broadleaf forests, roadsides, riversides, and natural pastures. Five of the habitat types were found close to each pair of apiaries, while broad-leaf forest was close to only two (apiaries A-B and C-D) and in the third area (apiaries E–F) the only riverside vegetation was present. In total flowers were identified and counted in 24 sites, as six types of vegetation close to each pair of apiaries and then a site next to each apiary. In each site an area of 200 m^2^ was established (14.5 m × 14.5 m or 100 m × 2 m). Additionally, we assessed the flowering plants close to each apiary, along the edge of a field if the apiary was placed next to a field, or along a road if the apiary was located in forest. For these we established areas of 100 m × 2 m (200 m^2^). All flowering plants within the area were identified based on literature on site^63^.

To assess the plants used for nectar foraging we collected a spoonful (approximately 15 g) of newly covered honey from three frames from each hive, to cover the diversity of honey at a time. The honey was collected by a DNA-clean spoon into one DNA-free 50 ml tube (Sarstedt AG & Co. KG, Germany), pooling the sample from the three frames. DNA-free spoons were prepared by washing with detergent and incubation for 5 h in + 200 °C. During the sampling in August another honey sample was collected to assess plant metabolites in honey. For this the pooled sample was collected the same way except sampling the spoonful from one newly covered honey frame and two frames with older honey, to get a sample representing the latter part of season more comprehensively. To identify which flowering species honeybees collect pollen from, in comparison to nectar, we collected a beebread sample from each hive at the same time points. Twenty cells of beebread were sampled from three frames, resulting in pooled sample of sixty beebread cells, to cover the diversity of stored pollen in a hive at a time. The beebread from a cell was sampled by pushing a plastic straw to the bottom of a cell and twisting, thus including all or most of the beebread in a cell. The samples were frozen immediately over dry ice in the field. All the samples were stored frozen in − 20 °C before further processing.

### Sample numbers

At the beginning of the sampling there were 43 hives from which we sampled, but unfortunately nearly one third of the colonies were left without a queen (queen had died or swarmed) before the second sampling, so 30 hives remained in July and 29 in August. In total we collected 99 honey samples and 90 beebread samples, as we were not able to get a proper sample. At the first sampling in June, eleven of the hives had no covered honey yet, so instead we sampled partially processed nectar as the honey sample.

### Sample preprocessing for DNA extraction

Before extracting the DNA, the samples were preprocessed. For honey, the sample collected from three frames was mixed and 10 g of honey was diluted to 30 ml of DNA clean water in a 50 ml tube. The honey was let to dissolve into the water for 30 min in 60°. The samples were then centrifuged at 8000 G for 60 min, after which most of the supernatant was discarded and the pellet was transferred to a 2 ml tube. The 2 ml tube was further centrifuged at 11,000 G for 5 min and the remaining supernatant was removed.

For beebread, the beebread was first extracted from the straw and weighed with a precision scale. The sample was then diluted in double distilled water with a 2:1 water-beebread weight ratio and mixed with magnetic stirrer for 10 min to produce a homogenized suspension. 100 µl of beebread suspension per sample was collected into a 2 ml microcentrifuge tube. The 2 ml tube was centrifuged at 16,873 G for 3 min and the supernatant was removed.

All the preprocessed samples were stored in freezer until DNA extraction.

### DNA extraction, amplifications, and sequencing

QIAamp DNeasy plant mini kit (Qiagen, Netherlands) was used to extract DNA with adapted manufacturer protocols. For the honey samples, the pellet was resuspended in 400 µl of buffer AP1, and then 4 µl RNase, 4 µl proteinase K (20 mg/ml) and one 3 mm tungsten carbide bead was added to each sample tube. The sample was disrupted 2 × 2 min 30 Hz (Mixer Mill MM 400, Retsch, Germany). DNA extraction then followed the protocol with the exception of skipping the QIAshredder column step and finally the DNA was eluted to 50 μl of elution buffer.

For beebread samples, the pellet was resuspended in 400 µl of buffer AP1 with two 5mm metal beads and disrupted 2 × 2 min 30 Hz (TissueLyser II, Qiagen, Netherlands). Incubation with buffer AP1 and 4 µl RNase A was done in 65 °C for 30 min, inverting tubes twice during incubation. Manufacturer protocol was thereafter followed, except 100 µl final elution volume was used with a single centrifugation step. With the extraction of each sample type, 2–3 DNA extract controls were included. We only used DNA-free tubes, pipet tips and PCR plates as well as DNA-free water.

The initial amplifications were done with a total volume of 10 μl, each containing 5 μl MyTaq Red Mix (Bioline, London, UK), 1.3 μl DNA-free water, 0.3 μM of each primer and 3 μl of DNA extract. To amplify a partial ITS2 region from both honey and beebread samples, we used the plant-targeted primers with a tag to attach the index in the second PCR, tagF_ITS2-F and tagR_ITS2-R (tcgtcggcagcgtcagatgtgtataagagacagATGCGATACTTGGTGTGAAT and gtctcgtgggctcggagatgtgtataagagacagTCCTCCGCTTATTGATATGC, respectively, tag shown in lower case and annealing primer in upper case^64, 65^). PCR cycling conditions were as follows. The initial denaturation was for 3 min at 95 °C, followed by 28 cycles of 30 s 95 °C (denaturation), 30 s 55 °C (annealing), 30 s 72 °C (extension), and ending with final extension for 7 min at 72 °C. To minimize initial bias of amplification, each reaction was carried out as two replicates. All the amplicons were checked on agarose gel and imaged to check the reaction had worked and the DNA and PCR controls were clean. The PCR replicates were combined before library-PCR as 1.3 μl of each PCR product replicate. Illumina‐specific adapters and unique dual‐index combinations for each sample was used ^66^. The library PCR had a total volume of 10 μl, each containing 5 μl MyTaq Red Mix (Bioline, London, UK), 0.3 μM of reverse primer, 0.3 μM of forward primer and 2.6 μl of the locus-specific combined 1st PCR product. PCR cycling conditions were as follows, the same for all gene regions for the library PCR. Starting with 4 min 95 °C to denature, followed by 15 cycles of 20 s 98 °C, 15 s 60 °C and 30 s 72 °C, and ending with 3 min 72 °C. DNA libraries were pooled per gene region and per 96 samples, and concentrated using a SPRI bead protocol. The concentrated pooled sample was loaded on 1% agarose gel (Agarose tablets + TAE) and run with 90 V for 120 min. The target bands were cut on UV light and the pooled sample was cleaned from gel with the PCR and Gel CleanUp Kit (Macherey–Nagel), diluted in 2 × 20 μl of the elution buffer provided in the kit. The DNA concentration of the cleaned pools were measured with Qubit 2.0 (dsHS DNA Kit, ThermoFisher Scientific).

The pools of 96 samples were combined in equimolar ratios and sequenced in two MiSeq sequencing runs (including other libraries also) with v3 chemistry with 600 cycles and 2 × 300 bp paired-end read length.

### Bioinformatics

The bioinformatic processing (following^67, 68^) firstly involved truncating the reads to 240 bp. This was done to cut off lower quality ends before merging the paired ends for each gene region using VSEARCH^69^ with a maximum of 80 differences allowed for overlap and a minimum assembly length of 150 bp. The merged reads were quality controlled by fastq_maxee, with maxee = 3. Primers were removed using cutadapt with a maximum of 0.2 error rate for primers, and reads were kept with minimum length of 100 bp after primer removal. The merged and quality-controlled reads were only retained if they contained the expected primers at each end. The reads were then dereplicated into uniques and singletons were removed. The reads were denoised to zero-radius operational taxonomic units (ZOTU) using with unoise3 with USEARCH^70^. A ZOTU table was built and the taxonomic assignation of ZOTUs was done by comparison against an ITS2 reference database from PLANTiTS^71^, accessed 21.3.2022 with VSEARCH. We consider here the taxonomic assignments as they resulted from the analyses without correcting based on species distributions, although some of the genus assignments might not be correct, e.g., *x-Amelasorbus* is a hybrid genus, and most likely the sequences assigned to it would originate from *Sorbus* in Finland.

To remove possible misassigned reads and false positives, due to contamination, we further filtered the reads in ZOTUs (following e.g.^72, 73^). As small numbers of reads were found in all controls, reads were removed for each ZOTU, from each sample if they were less reads in the sample than the maximum number of reads from the DNA extraction or PCR negative controls for the ZOTU. After taxonomic assignation, taxa with less than 0.05% of the total read number of that sample were removed, as well as taxa with less than 10 reads were removed. Samples with fewer than 5000 reads were removed to omit samples with shallow sequencing.

### Statistical methods

We calculated the relative read abundances (RRA) of each plant genus per sample ^32^, and RRA data was used for the analyses. We ran the analyses also using presence-absence (PA) data. Results from PA-analyses are in general agreement with the RRA-results and are available in the supplementary information. Statistical methods were implemented and figures generated in R version 4.2.2 ^74^, except for the specialized metabolite analyses (see below). *P* values of < 0.05 were considered statistically significant.

To identify the difference in the selection of floral resources by bees to produce honey or beebread, we first tested the multivariate homogeneity of the dispersion of groups between honey and beebread (PERMDISP, R function “betadisper” from the vegan package^75^, using the Hellinger-transformed data at genus-level). Second, we quantify the difference in plant genus composition between beebread and honey using a permutational analysis of variance (PERMANOVA, with the “adonis2” function of the vegan package^75^). As adonis2 does not allow random effects, the terms site, apiary, and hive were included in the model formula as fixed effects to account for pseudoreplication following the structure:

$${\text{adonis}}\;({\text{Hellinger}}\_{\text{aboundance}}\,\sim \,{\text{site}}/{\text{apiary}}/{\text{hive}}\, + \,{\text{sample}}\_{\text{type}}).$$

To describe how honey and beebread samples differ and group into distinct clusters based on their composition of plant genera, we applied nonmetric multidimensional scaling (NMDS, with function “metaMDS” from package vegan^75^). NMDS was further used to illustrate the temporal effects on the samples as well as comparison with metabolite datasets, for which pooled honey sample data from July and August were used. NMDS analyses were performed with Hellinger transformed data.

We analyzed the number of genera in each sample type (honey or beebread) with linear models, using time point as explanatory variable and hive as random variable, with function “lmer” from package lme4^76^. Pairwise comparisons for time points were made using emmeans-package^77^ with Tukey p-value adjustment. To analyze the proportion of shared and non-shared genera within the paired honey-beebread comparisons a binomial generalized linear model with logit-link function was used (using “glmer” function from package lme4^76^), again using time point as explanatory variable and hive as random variable. Model assumptions were checked visually and with the R package DHARMa^78^. To identify which plant genera are the most associated with the honey and beebread samples, we used the Indicator Species Analysis (IndVal, with function “multipatt” from the package indicspecies^79^, using 999 permutations).

To test the contribution of variables affecting floral choices, we used partial redundancy analysis (RDA) using Hellinger distances. The model follows the structure:

$${\text{RDA}}\;({\text{Hellinger}}\_{\text{abundance}}\,\sim \,{\text{sample}}\;{\text{type }}*{\text{ time}}\, + \,{\text{conditional}}\left( {{\text{site}}*{\text{apiary}}*{\text{hive}}} \right).$$

As with the PERMANOVA model, the RDA controls the design variables experimentally to account for pseudo-replication. The model was also tested with the sequencing read depth, which appeared to decrease the observed conditional variation by approximately 5% (Supplementary Table S8). Constrained ordination was tested by ANOVA-type permutation test in the vegan package. The variation associated with temporal, spatial and methodological variables were quantified by variation partitioning with the function “varpart” from the vegan package^75^.

### Extraction of plant specialized metabolites

Specialized metabolites were purified from honey samples from 27 hives. First, five grams of honey was measured into 50 ml centrifuge tubes in three replicates. Two different purification methods were employed to remove sugars and to recover as wide a variety of specialized metabolites as possible. Purification method 1 followed a method mainly directed on the enrichment of flavonoids, i.e., medium-polar metabolites^80^, with slight modifications. 15ml of water, adjusted to pH 2 with HCl, was mixed with the honey and stirred with magnetic stirrer for 15 min until completely fluid. Samples were then centrifuged 3220 G, 25 °C, 1 min to remove particles. The supernatant was loaded on an Oasis 500 mg HLB cartridge (Waters, USA), preconditioned with 10 ml methanol, followed by 10 ml water (pH 2), allowed to equilibrate for 10 min, and washed with 10 ml pure water. Analytes were eluted with 5 ml of methanol into a 15 ml centrifuge tube. The eluent was removed under a stream of nitrogen. Prior to measurement, samples were reconstituted in 1ml of 0.1% formic acid/acetonitrile (70/30) containing 0.025 mg/ml indomethacin as internal standard (ISTD) and filtered through 0.45 µm PTFE filters.

Since purification method 1 would lead to the loss of alkaloids and very polar compounds during the washing step, samples were purified with method 2 which is based on Quick Easy Cheap Effective Rugged Safe (QuEChERS) protocol. This has been widely applied for enrichment of various trace compounds, among them for the analysis of alkaloids in honey^81^. First, 10 ml of water and 10 ml of acetonitrile was mixed in the 5 g honey sample and thoroughly shaken until mixed. Subsequently 8.2 g of MgSO_4_.7H_2_O and 1 g of NaCl were added. The mixture was shaken vigorously for 1 min and then centrifuged for 5 min at 2465 G, 25 °C, 5 min. The upper layer was filtered through 0.45 µm cellulose acetate filter, and 1ml was dried under a stream of nitrogen and redissolved in 100 µl of MeOH/H2O 1/1 containing 0.01 mg/ml benzanilide as ISTD.

Analyses were performed on a Dionex Ultimate 3000 UHPLC hyphenated with a Thermo QExactive Hybrid Quadrupole Orbitrap mass spectrometer that was equipped with an H-ESI II probe (Thermo Fisher Scientific). As a stationary phase, an Acquity UPLC® HSS T3 1.8 µm, 100 × 2 mm column protected by an Acquity UPLC® HSS T3 1.8 µm, 2.1 × 5 mm guard column (Waters) was used. Two different separation methods were applied for the sample sets prepared by the two different purification methods:

For samples purified with method 1, the mobile phase consisted of water + 0.1% HCOOH (solvent A) and acetonitrile + 0.1% HCOOH (solvent B). The column temperature was 40 °C, the flow rate was 0.45 ml/min, and the gradient was as follows: 0–15 min, 5–25% B in A; 15–22 min, 25–70% B in A; 22–25 min, 70–100% B in A; 25–26 min, 100% B; 26–26.3 min, 100–5% B in A; 26.3–32 min, 5% B in A. For samples purified with method 2, the mobile phase consisted of water (solvent A) and acetonitrile (solvent B). The column temperature was 40 °C and the flow rate was 0.4 ml/min. The gradient was as follows: 0–22 min, 10–72% B in A; 22–22.5 min, 72–100% B in A; 22.5–25 min, 100% B in A; 25–25.5 min, 100–10% B in A; 25.5–30 min, 10% B in A. Injection volume for both methods was 3 µl.

The mass spectrometer was run in the ESI negative mode for separation method 1 and in the positive mode for separation method 2. The MS parameters were as follows: Probe heater temperature was 350 °C, capillary temperature was 330 °C, sheath gas flow was 50 arbitrary units, auxiliary gas flow was 10 arbitrary units, capillary voltage was 3 kV in the negative mode and 3.5 kV in the positive mode, and S- lens RF level was 60. Scan range was *m/z* 100–1,500, and resolution was 70,000 (FWHM) for full MS and 17,500 (FWHM) for data dependent MS^2^ scans. During the first 1.0 min (separation method 1) and 0.9 min (separation method 2) of elution, the eluent bypassed the mass spectrometer, and no data were recorded in order to avoid contamination of the MS with high levels of carbohydrates that were expected to be still present in the samples despite the purification measures.

As blank samples, the solvents used for preparation of the samples were injected, and as QC samples, pooled samples were prepared from both sample types (purification method 1 and 2) by mixing 10 µl of replicate 1 of each sample. Blank samples were injected at the beginning, in the middle and at the end of each sequence, and the QC samples were injected at intervals of 10 runs.

### Data processing and evaluation for specialized metabolites

Raw analytical data were processed with Compound Discoverer 3.2 using the following parameters: Retention time window for spectra selection was 1–32 min for dataset 1 (acquired in the ESI negative mode with separation method 1) and 0.9–26 min for dataset 2 (acquired in the ESI positive mode with separation method 2). Retention time alignment was performed with adaptive curve model (maximum RT shift 2 min, maximum mass tolerance 10 ppm). For detecting and grouping unknown compounds, S/N threshold was 3, minimum intensity threshold was 5,000,000 for dataset 1 and 10,000,000 for dataset 2, and RT tolerance was 1 min. S/N threshold for gap filling was 20. The output, a data matrix consisting of retention time and intensity of every feature in every sample, was exported to MS Excel for further treatment. In both datasets, first, features derived from the analytical background were removed, and the peak areas of the ISTD in all samples were graphically compared in order to inspect the dataset for samples that had not been injected properly. On this basis, sample 23_2 was excluded from dataset 1, and sample 5_2 was excluded from dataset 2.

Then, all remaining peak areas were normalized to the peak area of the ISTD in the respective run. In order to remove unreliable features, means and relative standard deviation were calculated for all features detected in the pooled quality control samples. Features with a relative standard deviation above 33% were considered unreliable and removed from the datasets. These pretreated datasets were subjected to multivariate data analysis (MVDA) using SIMCA 17 (Sartorius). Prior to MVDA, data were log-transformed and pareto-scaled. For unsupervised MVDA, principal component analysis (HCA) and hierarchical clustering analysis (HCA) were used. For supervised MDVA, OPLS-DA models with three classes that correspond to the three apiary areas were constructed.

For tracking metabolites occurring at high level across all apiary areas, average peak areas per apiary area were calculated for each metabolite in the two datasets. Those peaks occurring with average peak areas > 200,000,000 (dataset 1) and > 750,000,000 (dataset 2) in all three apiary areas were subjected to peak annotation.

Annotation of discriminant and common metabolites was performed either comparing retention time or MS data with authentic reference compounds (ID level 1), or by comparing calculated molecular formula and MS/MS fragmentation pattern with literature or database data (ID level 2), or in case that no literature or database data were available, by theoretical interpretation of these data (ID level 3).

### Supplementary Information

Supplementary Information.

## Data Availability

The sequence datasets generated during the current study are available in the Sequence Read Archive repository, in the BioProject PRJNA889252 https://dataview.ncbi.nlm.nih.gov/object/PRJNA889252?reviewer=gfl7mn7q3mt60s70onkmbf0mfh for the review, once accepted, in https://www.ncbi.nlm.nih.gov/sra/PRJNA889252.
