# Supplementary material for: Honeybees’ foraging choices for nectar and pollen revealed by DNA metabarcoding
Source: Sci Rep. 2023 Sep 7;13:14753. doi: 10.1038/s41598-023-42102-4 (PMC10484984; doi:10.1038/s41598-023-42102-4)
Supplement: Supplementary file 1 — Supplementary Information. [file 41598_2023_42102_MOESM1_ESM.docx]

Honeybees’ foraging choices for nectar and pollen revealed by DNA metabarcoding

# Supplementary material

Matti Leponiemi, Dalial Freitak, Miguel Moreno-Torres, Eva-Maria Pferschy-Wenzig, Antoine Becker-Scarpetta, Mikko Tiusanen, Eero J. Vesterinen, Helena Wirta

**Table S1**: Relative read abundances of all the detected genera in different timepoints in total beebread and honey, shown as percentages.

| Time point | June | | July | | August | |
| --- | --- | --- | --- | --- | --- | --- |
| Sample type | Beebread | Honey | Beebread | Honey | Beebread | Honey |
| *Acer* | 0.5 % | < 0.1% |  |  |  |  |
| *Achillea* | < 0.1% |  |  |  |  |  |
| *Aegopodium* |  |  | < 0.1% | 1.8 % |  | 0.1 % |
| *Anemone* | < 0.1% | 0.3 % |  |  |  |  |
| *Angelica* | < 0.1% |  | < 0.1% | < 0.1% | 0.2 % | < 0.1% |
| *Anthriscus* | 3.2 % | 2.2 % |  | < 0.1% |  | < 0.1% |
| *Aronia* | 0.4 % |  |  |  |  |  |
| *Artemisia* |  |  |  |  | 5.9 % | 2.2 % |
| *Barbarea* |  | < 0.1% |  |  |  |  |
| *Betula* | 0.2 % | < 0.1% |  |  |  | 0.7 % |
| *Brassica* | 17.4 % | 28.0 % | 45.1 % | 38.1 % | 21.1 % | 33.9 % |
| *Calluna* | < 0.1% |  | < 0.1% |  | 17.3 % | 0.8 % |
| *Camelina* |  |  | 1.2 % | 0.5 % |  | 0.2 % |
| *Capsella* | < 0.1% | < 0.1% |  |  |  |  |
| *Carduus* |  |  |  |  | < 0.1% | < 0.1% |
| *Chamaenerion* |  |  | < 0.1% | 0.1 % | < 0.1% | 0.5 % |
| *Chelidonium* | 0.6 % | 0.3 % |  | < 0.1% |  |  |
| *Chenopodium* |  |  | < 0.1% |  |  | < 0.1% |
| *Cirsium* | < 0.1% |  |  |  | 1.4 % | 0.3 % |
| *Comarum* |  |  |  | < 0.1% |  | 0.7 % |
| *Convallaria* | 0.2 % |  |  |  |  |  |
| *Coriandrum* |  |  | < 0.1% |  | < 0.1% | 0.4 % |
| *Cornus* | < 0.1% |  |  |  |  |  |
| *Crataegus* | < 0.1% |  |  |  |  |  |
| *Fallopia* | < 0.1% |  |  |  |  |  |
| *Filipendula* |  |  | 16.5 % | 1.4 % | < 0.1% | 1.2 % |
| *Frangula* | < 0.1% | < 0.1% |  |  |  | < 0.1% |
| *Hieracium* |  |  |  |  | < 0.1% |  |
| *Hydrangea* |  |  |  |  | 2.2 % | 0.2 % |
| *Hylotelephium* |  |  |  |  | 0.1 % |  |
| *Impatiens* |  | 0.2 % |  |  | 2.0 % | 1.0 % |
| *Lamium* |  |  |  |  |  | < 0.1% |
| *Linaria* |  |  | < 0.1% |  | 5.1 % | 0.4 % |
| *Lupinus* | < 0.1% |  |  | < 0.1% |  |  |
| *Lythrum* |  |  |  |  | < 0.1% |  |
| *Malus* | 0.3 % | 0.6 % |  | < 0.1% |  |  |
| *Medicago* |  |  |  | < 0.1% | < 0.1% | 0.1 % |
| *Myosotis* | 0.2 % | 19.9 % |  | 7.5 % |  | 1.1 % |
| *Paeonia* | < 0.1% |  |  |  |  |  |
| *Persicaria* |  |  |  |  | 0.4 % | 0.2 % |
| *Phacelia* |  |  | 2.0 % |  | 2.3 % | < 0.1% |
| *Pisum* | < 0.1% |  | 12.9 % | 0.3 % | 5.7 % | 2.7 % |
| *Populus* |  | < 0.1% |  |  |  | 0.5 % |
| *Prunus* | 1.1 % | 2.7 % |  | < 0.1% |  |  |
| *Quercus* | < 0.1% | 0.4 % |  | < 0.1% |  | < 0.1% |
| *Ranunculus* | 2.4 % | 1.5 % |  | 0.3 % |  | 0.1 % |
| *Raphanus* |  | < 0.1% | < 0.1% | 0.1 % | 10.5 % | 2.6 % |
| *Rhododendron* | 2.2 % | 1.2 % |  |  | < 0.1% |  |
| *Rosa* |  | 1.4 % |  | < 0.1% |  | 0.2 % |
| *Rubus* | 0.8 % | 2.6 % | < 0.1% | 33.5 % |  | 21.3 % |
| *Rumex* | 0.1 % | < 0.1% |  | < 0.1% |  |  |
| *Salix* | 9.2 % | 22.9 % |  | 0.3 % | < 0.1% | 0.2 % |
| *Sambucus* | 1.6 % | 3.6 % |  | < 0.1% |  |  |
| *Scorzoneroides* |  |  |  |  | 0.5 % | < 0.1% |
| *Sinapis* |  |  | 1.8 % | 1.8 % | 0.4 % | 4.5 % |
| *Sonchus* |  | < 0.1% | < 0.1% |  | 12.0 % | 4.3 % |
| *Sorbus* | 55.2 % | 8.5 % | < 0.1% | 0.7 % | < 0.1% | 0.1 % |
| *Spiraea* | 0.6 % | < 0.1% |  | < 0.1% |  |  |
| *Syringa* | 0.3 % | < 0.1% |  | < 0.1% |  |  |
| *Taraxacum* | 0.1 % | 1.5 % |  | < 0.1% |  | < 0.1% |
| *Trifolium* | < 0.1% | < 0.1% | 18.0 % | 10.4 % | 10.6 % | 17.6 % |
| *Tripleurospermum* | < 0.1% |  | 0.3 % | 0.3 % | 1.9 % | 0.9 % |
| *Vaccinium* | 2.4 % | 1.7 % |  | 0.8 % |  | < 0.1% |
| *Veronica* |  | < 0.1% |  |  |  |  |
| *Vicia* | < 0.1% |  | 1.8 % | 1.4 % |  | 0.7 % |
| *Viola* |  | < 0.1% |  | 0.1 % | 0.1 % | 0.1 % |
| *x Amelasorbus* | 0.4 % | 0.1 % |  |  |  |  |
| Genera | 39 | 33 | 20 | 33 | 29 | 42 |


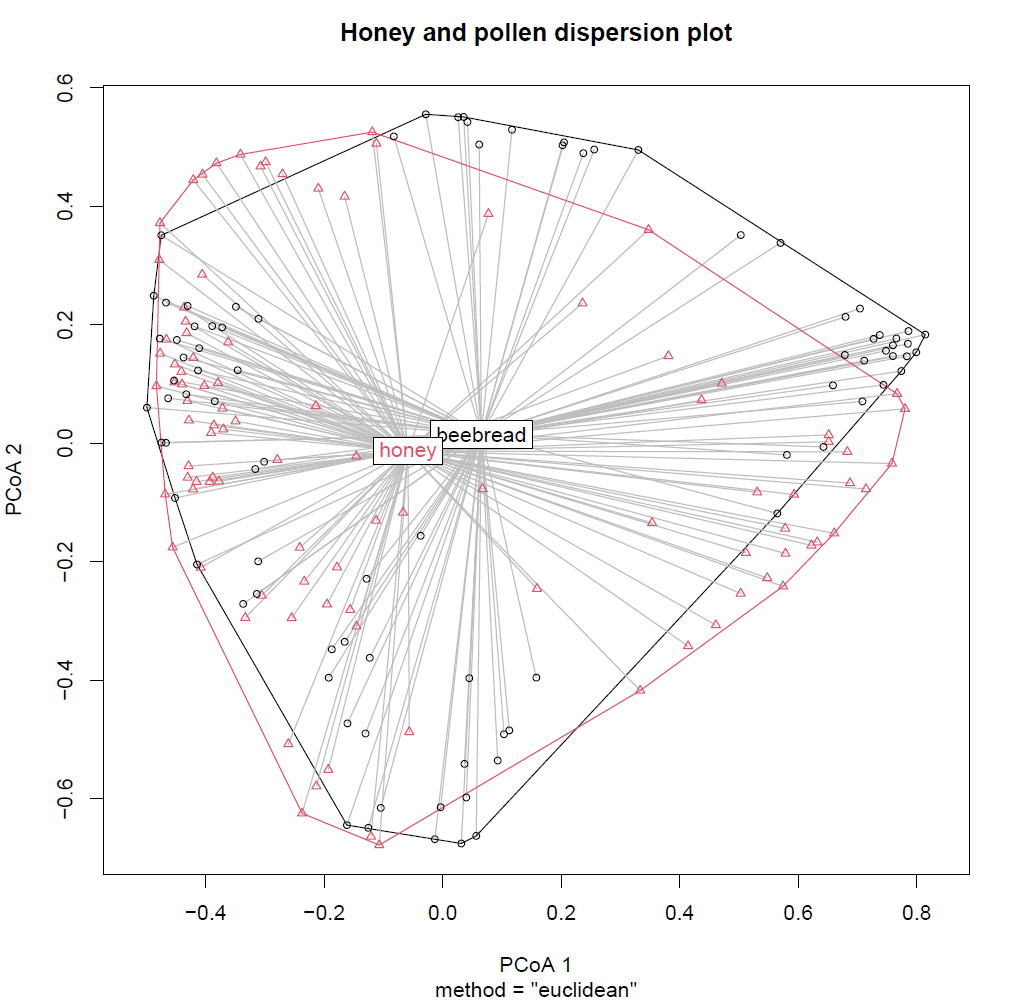


**Figure S1**: Dispersion plot showing the group variances for honey and beebread samples, based on the genera detected in each sample.

**Table S2**: Results for linear mixed model on genera detected in honey samples.

| Predictors | Estimates | CI | p |
| --- | --- | --- | --- |
| (Intercept) | 9.18 | 8.06 – 10.30 | **<0.001** |
| time [3] | 0.05 | -1.64 – 1.75 | 0.950 |
| time [4] | 3.36 | 1.63 – 5.09 | **<0.001** |

**Table S3**: Results for linear mixed model on genera detected in beebread samples.

| Predictors | Estimates | CI | p |
| --- | --- | --- | --- |
| (Intercept) | 9.19 | 8.46 – 9.93 | **<0.001** |
| time [3] | -3.37 | -4.46 – -2.28 | **<0.001** |
| time [4] | -0.89 | -2.05 – 0.27 | 0.130 |

**Table S4**: Results for linear mixed model on log transformed genera detected in the two sample types.

| Predictors | Estimates | CI | p |
| --- | --- | --- | --- |
| (Intercept) | 2.24 | 2.03 – 2.45 | **<0.001** |
| sample_type [beebread] | -0.24 | -0.34 – -0.13 | **<0.001** |

**Table S5**: Results for binomial generalized linear model shared genera in matched honey and beebread samples within hives.

| Predictors | Odds Ratios | CI | p |
| --- | --- | --- | --- |
| (Intercept) | 0.67 | 0.55 – 0.83 | **<0.001** |
| time [3] | 1.07 | 0.79 – 1.46 | 0.656 |
| time [4] | 0.77 | 0.57 – 1.05 | 0.103 |

**Table S6**: Genera detected as significant indicative species by IndVal for honey and beebread, using presence-absence-data instead of relative abundances as in the main text. In honey samples all the same genera were found indicative of honey samples as were based on the relative read abundances, and additionally the genera *Sorbus*, *Ranunculus* and *Viola*. In beebread the same genera were indicated except *Pisum* and *Cirsium*.

| **Honey** | **A (specificity)** | **B (fidelity)** | **stat** | **p.value** |
| --- | --- | --- | --- | --- |
| *Rubus* | 0.795 | 0.845 | 0.820 | 0.001 |
| *Myosotis* | 0.892 | 0.474 | 0.650 | 0.001 |
| *Sorbus* | 0.590 | 0.629 | 0.609 | 0.012 |
| *Vicia* | 0.689 | 0.485 | 0.578 | 0.002 |
| *Salix* | 0.594 | 0.505 | 0.548 | 0.041 |
| *Prunus* | 0.818 | 0.309 | 0.503 | 0.001 |
| *Taraxacum* | 0.854 | 0.268 | 0.478 | 0.001 |
| *Malus* | 0.659 | 0.289 | 0.436 | 0.026 |
| *Ranunculus* | 0.705 | 0.247 | 0.418 | 0.020 |
| *Chamaenerion* | 0.782 | 0.165 | 0.359 | 0.023 |
| *Rosa* | 1.000 | 0.124 | 0.352 | 0.001 |
| *Viola* | 0.878 | 0.082 | 0.269 | 0.048 |
| *Medicago* | 0.878 | 0.082 | 0.269 | 0.037 |
| *Populus* | 1.000 | 0.062 | 0.249 | 0.028 |
| *Comarum* | 1.000 | 0.062 | 0.249 | 0.031 |
| **Beebread** | **A (specificity)** | **B (fidelity)** | **stat** | **p.value** |
| *x Amelasorbus* | 0.671 | 0.380 | 0.505 | 0.005 |
| *Calluna* | 0.661 | 0.322 | 0.461 | 0.019 |
| *Rhododendron* | 0.690 | 0.253 | 0.418 | 0.019 |
| *Syringa* | 0.796 | 0.161 | 0.358 | 0.009 |
| *Crataegus* | 1.000 | 0.092 | 0.303 | 0.002 |
| *Aronia* | 1.000 | 0.069 | 0.263 | 0.010 |

**
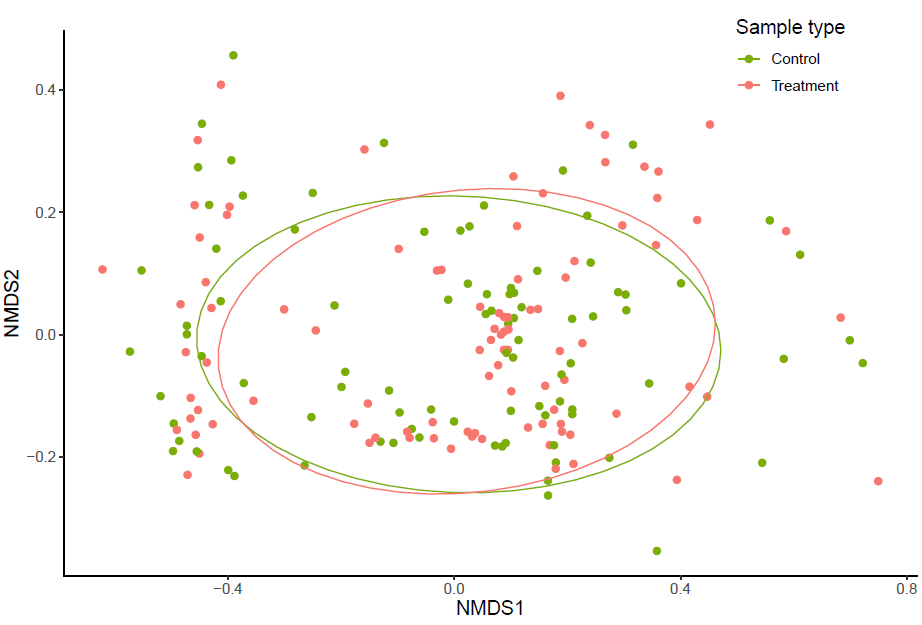
**

**Figure S2:** The composition of the communities within the two treatments by two dimensional NMDS (NMDS Stress 0.1469416), showing almost perfect overlap. PERMDISP and PERMANOVA indicate no treatment effect (PERMDISP, Groups, F=0.07, p=0.79, PERMANOVA Treatment F=0.33, p=0.95).

**
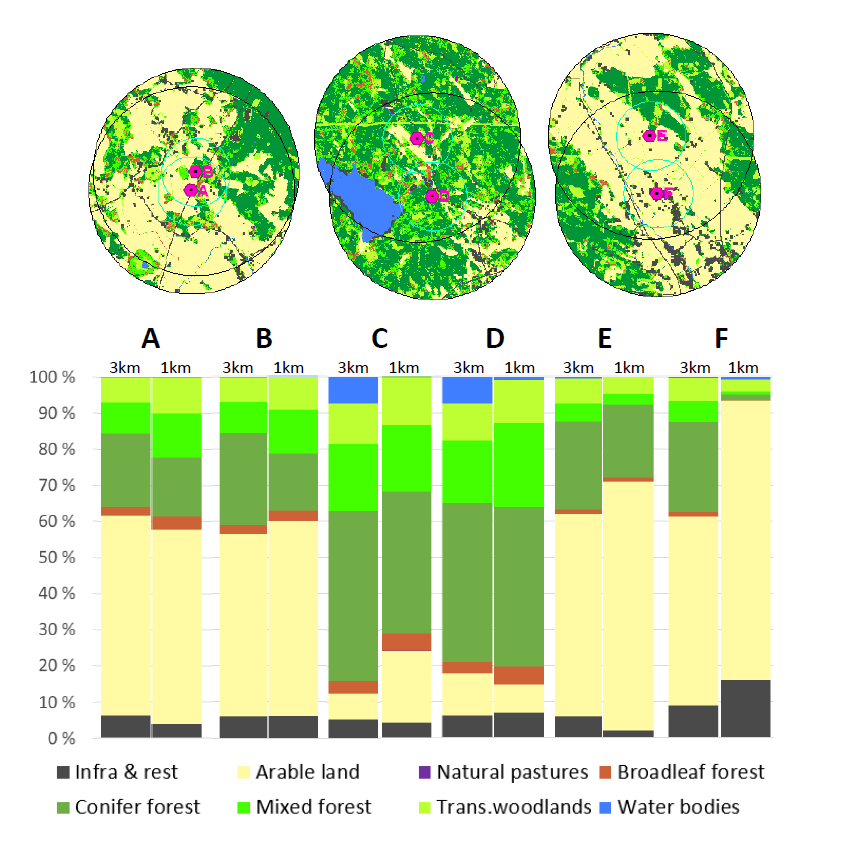
**

**Figure S3:** Map and proportions of land use of apiary areas. Map showing 3 km radius and 1km circle shown in cyan, and land use proportions within 3 km and 1 km radius of the apiaries.

**Table S8**: Alternative RDA model version results with sequencing depth, with variation partitioning results from varpart procedure. RDA model structure as: (Hellinger abundance ~ sample_type * time + read_depth + Conditional(area*apiary*hive)

| **Variables** | **df** | **Variance** | **F** | **P-value** |
| --- | --- | --- | --- | --- |
| sample_type | 1 | 0.045 | 26.731 | **0.001** |
| time | 2 | 0.145 | 42.774 | **0.001** |
| read_depth | 1 | 0.030 | 17.625 | **0.001** |
| sample_type:time | 2 | 0.052 | 15.204 | **0.001** |
| Residual | 135 | 0.229 |  |  |
| **Fractions** | **df** | **Adj. R^2^** |  |  |
| X1 = sample_type & time | 3 | 0.361 |  |  |
| X2 = Area, apiary & hive | 42 | 0.073 |  |  |
| X3 = read_depth | 1 | 0.056 |  |  |
| Residuals |  | 0.569 |  |  |

# Text S1: Specialized metabolites in honey

The CD-processed dataset 1 consisted of 1000 features. After removal of background and unreliable features, 771 features were subjected to MVDA. Dataset 2 initially consisted of 2925 features, 1851 of which were subjected to MVDA after removal of background and unreliable features.

In the PCA score scatter plot of complete dataset 1 (Fig. S4A) and dataset 2 (Fig. S5A) QC samples formed one tight cluster, indicating an excellent repeatability of the analytical method over the whole sequence. Also, the three replicate extracts prepared from each honey sample generally formed tight clusters, indicating a good reproducibility of the extraction methods. Only in dataset 1, in case of sample 41, replicate 2 was identified as an outlier and therefore removed from further analysis.

The PCA model generated from dataset 1 after removal of QC samples and sample 41_2 from the data matrix consisted of 21 principal components that altogether explained 95.6% of the observed variation (Fig. S4B). In the Hotelling´s T2 range line plot, no severe outliers were observable. Coloring the samples according to apiary indicated a trend to form clusters according to apiary for some, but not for all apiaries (Fig. S4B). When samples were colored according to apiary areas, samples from the areas AB and CD formed distinct clusters, and samples of area EF were scattered between these two clusters (Fig. S4C). This was supported by HCA based on this PCA model: in the dendrogram (Fig. S4D), two distinct major clusters were observed where apiary area AB samples differentially separated from apiary area CD samples. Apiary EF samples occurred in both major clusters.

The PCA model generated from dataset 2 consisted of 18 principal components that altogether explained 83.4% of the observed variation. Also in this dataset, samples from some apiaries formed clusters (Fig. S5B), and apiary area AB and CD samples again formed distinct clusters, with area EF samples scattered between them (Fig. S5C). In the HCA, again, two main clusters were observed, with area CD samples located in one of them, and the majority of area AB samples (except for hive 36 and 47 samples located in the other one (Fig. S5D).


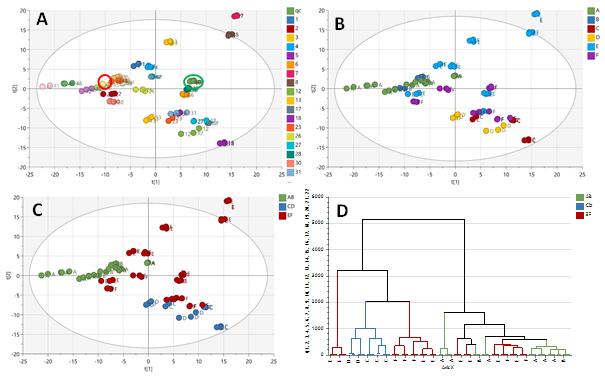


**Figure S4**: Unsupervised MVDA of dataset 1. **A:** PCA score scatter plot [t1]/[t2] of all samples excl. 23_2 (21 principal components, R2X (cum) = 0.956, Q2 (cum)= 0.889; R2X [t1] = 0.312, R2X [t2]= 0.175. Coloring is according to hive. Red circle: sample 41_2; green circle: QC samples **B:** PCA score scatter plot [t1]/[t2] of all samples excl. 23_2, 41_2 and QC samples; (27 principal components, R2X (cum) = 0.981, Q2 (cum)= 0.915; R2X [t1] = 0.304, R2X [t2]= 0.184. Coloring is according to apiary. **C:** PCA score scatter plot [t1]/[t2] as in **B**. Coloring is according to apiary area. **D:** HCA dendrogram corresponding to **C**.


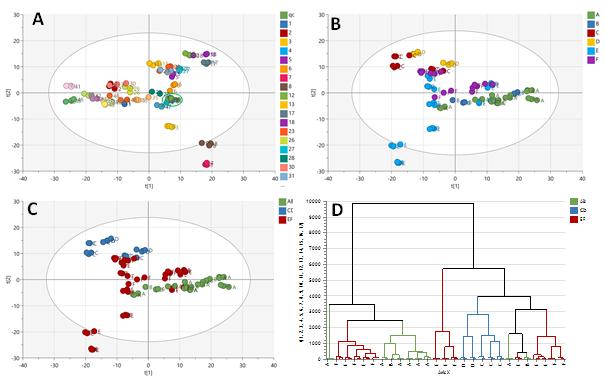


**Figure S5**: Unsupervised MVDA of dataset 2. **A:** PCA score scatter plot [t1]/[t2] of all samples (18 principal components, R2X (cum) = 0.840, Q2 (cum)= 0.668; R2X [t1] = 0.217, R2X [t2]= 0.113. Coloring is according to hive. Green circle: QC samples **B:** PCA score scatter plot [t1]/[t2] of all samples excl. QC samples; (17 principal components, R2X (cum) = 0.834, Q2 (cum)= 0.658; R2X [t1]= 0.219, R2X [t2]= 0.117. Coloring is according to apiary. **C:** PCA score scatter plot [t1]/[t2] as in **B**. Coloring is according to apiary area. **D:** HCA dendrogram corresponding to **C**.

*Major metabolites common to all three apiary areas*

The distribution of the annotated commonly occurring major constituents in the samples from the three apiary areas is shown in Figures S6 and S7.

Many common constituents annotated from dataset 1 belonged to the compound class of mono- and sesquiterpenes. D1_c_2-D1_c_4 constituted structural isomers that were tentatively annotated as isomers of the iridoid glycoside deoxylamiol (Figure S6, Table S9, S10). D1_c_7 was annotated as the sesquiterpene derivative abscisic acid, an important plant hormone. Several other common constituents were annotated as aromatic (D1_c_1) or aliphatic carboxylic (D1_c_11) or dicarboxylic acids (D1_c_6 and D1_c_8). Finally, two constituents were annotated as tricoumaroylspermidine isomers that belong to the compound class of hydroxycinnamoylamides and frequently occur in pollen.

The common constituents annotated from data set 2 were highly consistent with the ones from data set 1 (Figure S7, Table S9, S10). From this data set, two deoxylamiol isomers (D2_c_3-4), five tricoumaroylspermidine isomers (D2_c_6-10) and again abscisic acid (D2_c_5) were annotated, as well as the vitamin pantothenic acid (D2_c_1) and a hydroxyquinolone isomer (D1_c_2).

**Table S9: Annotated honey constituents occurring at high levels across all apiary areas.** ID level [1,2]. **1**, confident 2D structure, identified by reference standard match (retention time and MS/MS); **2**, tentative annotation by comparison of structural formula and MS/MS fragmentation pattern with data from databases or literature. **3**, no data available in literature or databases; compound tentatively assigned on the basis of the MS/MS fragmentation pattern (theoretical interpretation and/or comparison to related compounds) (annotation details, see Table S10). Mean peak areas in the respective apiary areas are colored with a threepartite color scale (red = low, white= medium, green= high abundance), boxplots illustrating the distribution of the metabolites in the apiary areas are depicted in Figure S6 and S7. Statistical comparison of peak areas between apiary areas was performed by Kruskal-Wallis One Way Analysis of Variance on Ranks* followed by pairwise multiple comparison with Dunn´s method** (p<0.05 marked in gray)

| **compound** | **RT [min]** | **neutral monoisotopic mass** | **mean peak area (AB)** | | **mean peak area (CD)** | | **mean peak area (EF)** | | **p-value (all apiary areas)*** | | **p-value (CD vs AB)**** | | **p-value (CD vs EF)**** | | **p-value (EF vs AB)**** | | **annotation^IDlevel^** | | | **compound class** | | |
| --- | --- | --- | --- | --- | --- | --- | --- | --- | --- | --- | --- | --- | --- | --- | --- | --- | --- | --- | --- | --- | --- | --- |
| **Dataset 1** | | | | | | | | | | | | | | | | | | | | | |  |
| **D1_c_1** | 7.35 | 166.0627 | 9.42E+08 | | 1.03E+09 | | 4.61E+08 | | 0.015 | | 0.653 | | 0.037 | | 0.083 | | phenyllactic acid^1^ | | | carboxylic acid | | |
| **D1_c_2** | 10.11 | 362.1577 | 8.39E+08 | | 3.82E+08 | | 6.68E+08 | | 0.001 | | <0.001 | | 0.023 | | 0.293 | | deoxylamiol isomer 1^2^ | | | monoterpene glycoside | | |
| **D1_c_3** | 10.34 | 362.1577 | 6.47E+08 | | 3.43E+08 | | 6.17E+08 | | 0.003 | | 0.007 | | 0.004 | | 1.000 | | deoxylamiol isomer 2^2^ | | | monoterpene glycoside | | |
| **D1_c_4** | 10.88 | 362.1577 | 1.24E+09 | | 5.85E+08 | | 1.01E+09 | | <0.01 | | <0.01 | | 0.02 | | 0.358 | | deoxylamiol isomer 3^2^ | | | monoterpene glycoside | | |
| **D1_c_5** | 11.42 | 362.1577 | 1.82E+09 | | 9.04E+08 | | 1.59E+09 | | 0.001 | | <0.001 | | 0.015 | | 0.657 | | deoxylamiol isomer 4^2^ | | | monoterpene glycoside | | |
| **D1_c_6** | 14.30 | 200.1047 | 1.46E+09 | | 1.03E+09 | | 1.36E+09 | | 0.058 | |  | |  | |  | | camphoric acid or decenedoic acid^2^ | | | dicarboxylic acid | | |
| **D1_c_7** | 14.86 | 264.1360 | 2.02E+08 | | 1.66E+09 | | 3.87E+08 | | 0.007 | | 0.005 | | 0.115 | | 0.378 | | absciscic acid isomer^2^ | | | sesquiterpene | | |
| **D1_c_8** | 15.54 | 202.1203 | 1.64E+09 | | 1.06E+09 | | 1.55E+09 | | 0.035 | | 0.042 | | 0.068 | | 1.000 | | sebacic acid isomer^2^ | | | dicarboxylic acid | | |
| **D1_c_9** | 17.05 | 583.2683 | 6.13E+08 | | 1.22E+09 | | 1.18E+09 | | 0.01 | | 0.051 | | 1.000 | | 0.016 | | tricoumaroylspermidine isomer 1^2^ | | | hydroxycinnamoylamide | | |
| **D1_c_10** | 17.27 | 583.2683 | 3.68E+08 | | 9.44E+08 | | 6.43E+08 | | 0.03 | | 0.028 | | 0.505 | | 0.287 | | tricoumaroylspermidine isomer 2^2^ | | | hydroxycinnamoylamide | | |
| **D1_c_11** | 17.98 | 328.2249 | 2.23E+08 | | 6.67E+08 | | 5.43E+08 | | 0.008 | | 0.015 | | 1.000 | | 0.037 | | trihydroxyoctadecadienoic acid FA18:2+3O isomer ^2^ | | | carboxylic acid | | |
|  |  |  | |  | |  | |  | |  | |  | |  | |  | |  |  |  |  |  |
| **Dataset 2** | | | | | | | | | | | | | | | | | | | | | |  |
| **D2_c_1** | 1.16 | 219.1107 | 8.E+08 | | 1.E+09 | | 8.E+08 | | 0.068 | |  | |  | |  | | panthotenic acid^2^ | | | vitamin | | |
| **D2_c_2** | 2.08 | 145.0528 | 6.E+09 | | 3.E+09 | | 7.E+09 | | 0.268 | |  | |  | |  | | hydroxyquinoline or isomer^2^ | | | quinoline | | |
| **D2_c_3** | 4.31 | 362.1577 | 2.E+09 | | 8.E+08 | | 2.E+09 | | 0.003 | | 0.006 | | 0.004 | | 1 | | deoxylamiol isomer 1^2^ | | | monoterpene glycoside | | |
| **D2_c_4** | 4.58 | 362.1557 | 9.E+08 | | 9.E+08 | | 9.E+08 | | 0.854 | |  | |  | |  | | deoxylamiol isomer 2^2^ | | | monoterpene glycoside | | |
| **D2_c_5** | 6.36 | 264.1362 | 1.E+09 | | 9.E+09 | | 2.E+09 | | 0.095 | |  | |  | |  | | abscisic acid ^2^ | | | sesquiterpene | | |
| **D2_c_6** | 8.47 | 583.2682 | 3.E+09 | | 7.E+09 | | 6.E+09 | | <0.001 | | 0.005 | | 1 | | 0.001 | | tricoumaroylspermidine isomer 1^2^ | | | hydroxycinnamoylamide | | |
| **D2_c_7** | 8.69 | 583.2682 | 3.E+09 | | 7.E+09 | | 6.E+09 | | 0.008 | | 0.008 | | 0.419 | | 0.116 | | tricoumaroylspermidine isomer 2^2^ | | | hydroxycinnamoylamide | | |
| **D2_c_8** | 8.74 | 583.2682 | 9.E+08 | | 2.E+09 | | 1.E+09 | | 0.038 | | 0.044 | | 0.822 | | 0.211 | | tricoumaroylspermidine isomer 3^2^ | | | hydroxycinnamoylamide | | |
| **D2_c_9** | 8.84 | 583.2682 | 8.E+08 | | 2.E+09 | | 1.E+09 | | 0.027 | | 0.028 | | 0.608 | | 0.217 | | tricoumaroylspermidine isomer 4^2^ | | | hydroxycinnamoylamide | | |
| **D2_c_10** | 9.04 | 583.2682 | 9.E+08 | | 3.E+09 | | 1.E+09 | | 0.02 | | 0.016 | | 0.201 | | 0.514 | | tricoumaroylspermidine isomer 5^2^ | | | hydroxycinnamoylamide | | |

**Table S10**: **Details for annotation of metabolites occurring at high levels across all apiary areas.** Compound numbering as in Table S9. ID level [1,2]: **1**, confident 2D structure, identified by reference standard match (retention time and MS/MS); **2**, tentative annotation by comparison of structural formula and MS/MS fragmentation pattern with data from databases or literature. **3**, no data available in literature or databases; compound tentatively assigned on the basis of the MS/MS fragmentation pattern (theoretical interpretation and/or comparison to related compounds).

| **compound** | **RT [min]** | ***m/z* (experimental)** | **neutral monoisotopic mass** | **calculated neutral formula** | | **Δ (ppm)** | | **MS/MS fragments** | **ID^ID level^** | **Annotation source** | **Detected in bee product (example)** | **Compound class** |
| --- | --- | --- | --- | --- | --- | --- | --- | --- | --- | --- | --- | --- |
| **Dataset 1 (ESI negative mode ionization); Common metabolites D1_c_1- D1_c_11** | | | | | | | | | | | | |
| **D1_c_1** | 7.35 | 165.0554 | 166.06277 | C_9_H_10_O_3_ |  | | 165.0554 (35) 147.0450 (100) 119.0500 (35) | | phenyllactic acid^1^ | reference compound | honey [3] | carboxylic acid |
| **D1_c_2** | 10.11 | 361.1502 | 362.15775 | C_16_H_26_O_9_ | 4.54 | | 223.0976 (5) 199.0974 (45) 155.1076 (100) 137.0969 (15) 113.0244 (5) 101.0243 (15) | | deoxylamiol isomer 1^2^ | literature [4] |  | monoterpene glycoside |
| **D1_c_3** | 10.34 | 351.1502 | 362.15775 | C_16_H_26_O_9_ | 2.57 | | 199.0976(55) 181.0871 (10) 155.1077 (100) 137.0972 (25)101.0244 (15) | | deoxylamiol isomer 2^2^ | literature [4] |  | monoterpene glycoside |
| **D1_c_4** | 10.88 | 361.1502 | 362.15776 | C_16_H_26_O_9_ | 2.5 | | 223.0977 (10) 199.0976 (60) 155.1077 (100) 137.0971 (25) 101.0244 (15) | | deoxylamiol isomer 3^2^ | literature [4] |  | monoterpene glycoside |
| **D1_c_5** | 11.42 | 361.1502 | 362.15778 | C_16_H_26_O_9_ | 2.5 | | 361.1501 (3) 301.1303 (5) 223.0975 (25) 199.0977 (50) 179.1077 (10) 155.1077 (100) 137.0972 (20) 101.0244 (10) 59.0139 (5) | | deoxylamiol isomer 4^2^ | literature [4] |  | monoterpene glycoside |
| **D1_c_6** | 14.30 | 199.0974 | 200.10474 | C_10_H_16_O_4_ | 2.41 | | 199.0975 (10) 155.1076 (100) 137.0970 (5) | | camphoric acid or decenedoic acid^2^ | literature [5,6] | camphoric acid in honey ^[7]^; decenedioic acid in royal jelly ^[5]^  and honey ^[8]^ | dicarboxylic acid |
| **D1_c_7** | 14.86 | 263.1286 | 264.13603 | C_15_H_20_O_4_ | 4.34 | | 263.1286 (5) 219.1391 (100) 204.1155 (95) 201.1286 (35) 189.0923 (10) 163.0760 (20) 151.0764 (70) 139.0764 (40) 125.0607 (30) 97.0293 (15) | | absciscic acid isomer^2^ | MoNA VF-NPL-QEHF000236 | honey (frequent) [9,10] | sesquiterpene |
| **D1_c_8** | 15.54 | 201.1131 | 202.12039 | C_10_H_18_O_4_ | 3.21 | | 201.1131 (85) 183.1024 (65) 139.1127 (100) | | sebacic acid isomer^2^ | MoNA PT203390 | royal jelly [5] and honey [8] | dicarboxylic acid |
| **D1_c_9** | 17.05 | 582.2606 | 583.26834 | C_34_H_37_O_6_N_3_ | 4.2 | | 582.2614 (15) 462.2030 (20) 342.1457 (30) 316.1664 (10) 145.0293 (25) 119.0501 (100) | | tricoumaroylspermidine isomer 1^2^ | literature [11] | rape bee pollen [11] | hydroxycinnamoylamide |
| **D1_c_10** | 17.27 | 582.2607 | 583.26835 | C_34_H_37_O_6_N_3_ | 1.3 | | 582.261 (20) 462.2032 (20) 342.1456 (25) 316.1664 (10) 145.0294 (25) 119.0500 (100) | | tricoumaroylspermidine isomer 2^2^ | literature [11] | rape bee pollen [11] | hydroxycinnamoylamide |
| **D1_c_11** | 17.98 | 327.2175 | 328.22491 | C_18_H_32_O_5_ | 1.41 | | 327.2176 (100) 291.1967 (10) 229.1443 (30) 211.1339 (45) 171.1025 (65) 137.0971 (10) 97.0658 (10) 85.0295 (25) | | trihydroxyoctadecadienoic acid FA18:2+3O isomer ^2^ | MoNA VF-NPL-QTOF000050 |  | carboxylic acid |
| **Dataset 2 (ESI positive mode ionization); Common metabolites D2_c_1- D2_c_10** | | | | | | | | | | | | |
| **D2_c_1** | 1.16 | 220.118 | 219.1107 | C_9_H_17_NO_5_ | | -0.41 | | 220.1181 (30) 202.175 (35) 184.0969 (25) 142.0864 (10) 124.0759 (20) 116.0345 (15) 103.0759 (10) 98.0243 (15) 90.0555 (100) 85.0655 (10) 72.0452 (15) | panthotenic acid^2^ | MoNA RP012302 | honey [12] | vitamin |
| **D2_c_2** | 2.08 | 145.0528 | 145.0528 | C_9_H_7_ON | | 1.3 | | 146.0602 (100) 121.9665 (5) 105.0705 (3) 87.0448 (3) | hydroxyquinoline or isomer^2^ | MzCloud Reference-2633 | honey [13] | quinoline |
| **D2_c_3** | 4.31 | 363.1653 | 362.1577 | C_16_H_26_O_9_ | | 1.05 | | 363.1883 (15) 325.1434 (15) 309.1347 (20) 224.0716 (10) 201.1390 (20) 183.1030 (20) 165.0910 (30) 155.1071 (35) 137.0964 (100) 127.0393 (25) 121.0393 (35) 109.1019 (40) 95.0862 (95) 93.0706 (45) | deoxylamiol isomer 1^2^ | literature [4] |  | monoterpene glycoside |
| **D2_c_4** | 4.58 | 385.1473 [M+Na]+ | 362.1557 | C_16_H_26_O_9_ | | 1.13 | | 385.1472 (100) 367.1368(5) 223.0929 (3) | deoxylamiol isomer 2^2^ | literature [4] |  | monoterpene glycoside |
| **D2_c_5** | 6.36 | 287.1258 [M+Na]+ | 264.1362 | C_15_H_20_O_4_ | | -0.59 | | 287.1255 (100) 247.0919 (10) 228.3454 (3) 201.1288 (5) 173.1332 (5) 161.3284 (3) 91.8670 (5) | abscisic acid ^2^ | MzCloud reference-158 | honey (frequent) [9,10] | sesquiterpene |
| **D2_c_6** | 8.47 | 584.275 | 583.2682 | C_34_H_37_O_6_N_3_ | | -1.88 | | 438.2385 (75) 420.2272 (15) 292.2016 (30) 275.1754 (15) 218.1177 (5) 204.1021 (60) 147.0441 (100) 119.0495 (15) | tricoumaroylspermidine isomer 1^2^ | literature [14] | rape bee pollen [11] | hydroxycinnamoylamide |
| **D2_c_7** | 8.69 | 584.275 | 583.2682 | C_34_H_37_O_6_N_3_ | | -1.88 | | 438.2385 (75) 240.2280 (20) 292.2018 (25) 218.1177 (5) 204.1021 (50) 147.0441 (100) 119.0494 (10) | tricoumaroylspermidine isomer 2^2^ | literature [14] | rape bee pollen [11] | hydroxycinnamoylamide |
| **D2_c_8** | 8.74 | 584.2751 | 583.2682 | C_34_H_37_O_6_N_3_ | | -1.66 | | 584.2783 (5) 438.2390 (65) 420.2284 (15) 292.2022 (25) 218.1177 (10) 204.1023 (55) 147.0442 (100) | tricoumaroylspermidine isomer 3^2^ | literature [14] | rape bee pollen [11] | rape bee pollen [11] |
| **D2_c_9** | 8.84 | 584.2751 | 583.2682 | C_34_H_37_O_6_N_3_ | | -1.66 | | 584.2721 (5) 438.2388 (85) 420.2274 (25) 292.2025 (30) 275.1758 (15) 218.1184 (5) 204.1022 (80) 147.0441 (100)119.0494 (10) | tricoumaroylspermidine isomer 4^2^ | literature [14] | rape bee pollen [11] | hydroxycinnamoylamide |
| **D2_c_10** | 9.04 | 584.2755 | 583.2682 | C_34_H_37_O_6_N_3_ | | -0.94 | | 584.2759 (5) 438.2389 (60) 420.2280 (30) 292.2021 (20) 275.1752 (15) 218.1178 (10) 204.1021 (70) 147.0441 (100) 119.0493 (10) | tricoumaroylspermidine isomer 5^2^ | literature [14] | rape bee pollen [11] | hydroxycinnamoylamide |

**Figure S6**: Boxplots showing peak areas of major constituents occurring across all apiary areas annotated in dataset 1 (See Table S9 and S10). Solid line: median, dashed line: average.

**Figure S7**: Boxplots showing peak areas of major constituents occurring across all apiary areas annotated in dataset 2 (see Table S9 and S10). Solid line: median, dashed line: average.

*Metabolites discriminant between apiary areas*

In order to assess, which metabolites present in the samples are responsible for the trend to cluster according to apiary areas observed in PCA and HCA, OPLS-DA models were generated from both datasets. As can be seen in Figures S8 and S9, in the scatter plots t[1]/to[1] of both models, the samples were clearly separated according to apiary areas. CV-ANOVA indicated that both models were statistically good (CV-ANOVA p-value <0.05), and the response permutation test indicated a good predictive capability of both models (Fig. S8B, S8B): in both models, the computed R2Y(cum) and Q2(cum) values were in all cases significantly lower than the model values, indicating that none of the permutated models (n=100) performed better than the original ones [15,16]. For selection of variables that likely contribute to class separation, a combination of variable importance projection (VIP) scores and |p(corr)| was used [16]. Variables with VIP scores >1.2 and |p(corr)| values >0.5 were considered as relevant for class separation (Fig. S8C and S9C). 78 variables from dataset 1 and 170 variables from dataset 2 fulfilled these. The variables were sorted according to descending maximum peak area in the honey samples, and the major discriminant variables were annotated, either by comparison with authentic reference compounds, by comparison of their MS data with the literature or databases, or by theoretical interpretation of their MS/MS fragmentation patterns (Table S11 and S12).

The constituents that could be annotated are summarized in Table S11, which also contains their mean peak areas in the samples from the three apiary areas. Boxplots illustrating their occurrence in the samples of the three apiary areas are displayed in Figures S10 and S11.

From dataset 1, 12 major discriminant markers could be annotated that belonged to different compound classes. D1_d_1 which was particularly enriched in samples from apiary region CD, was identified as the phenyl glycoside salicin. D1_d_2 and _4 were tentatively annotated as two methyl oxopentanoic acid isomers. They occurred at higher levels in apiary EF samples. D1_d_3, predominantly occurring in apiary area CD samples, was annotated as the dicarboxylic acid isopropyl malic acid. A series of isomeric compounds that were more enriched in apiary region CD samples were tentatively annotated as monoterpene glycosides, most likely as isomers of the iridoid glycosides nepetaside (D1_d_5_and -6) and deoxycatalpol (D1_d_7-9), and D1_d_11, which was present at higher levels in samples from apiary regions AB and EF than in CD, was tentatively annotated as an isomer of deoxylamiol dihexoside, i.e. an iridoid with three sugar moieties. D1_d_10 that predominantly occurred in apiary region AB samples was annotated as a hydroxycinnamoylamide, namely dicoumaroylspermidine, and D1_d_12, more abundant in apiary region CD samples, was tentatively annotated as the flavonoid pinobanksin.

From dataset 2, 8 discriminant constituents occurring at higher levels were annotated. D2_d_1 was identified as the vitamin nicotinic acid, and D2_d_2 was tentatively annotated as the amino acid degradation product phenylethylamine. They occurred at higher levels in apiary regions CD and EF than in AB samples. D2_d_3 was assigned to a megastigmane glycoside, most likely vomifoliol. From dataset 2, a series of further hydroxycinnamoylamides was annotated, namely three dicoumaroyl-caffeoylspermidine isomers (D2_d_4-_6). D2_d_7 and _8 were identified as flavonoids, namely chrysin, and tetrochrysin or an isomer thereof. D2_d_4- _8 were more enriched in samples from apiary region CD than in the other samples.

**Figure S8:** OPLS-DA model for dataset 1 (all samples excl. 23_2, 41_2 and QC samples), classified by apiary area [2+6+0 principal components; R2X (cum)= 0.759, R2(cum) =0.99, Q2(cum)=0.978]. **A**: OPLS-DA score scatter plot t[1]/to[1], colored according apiary area (red: apiary area AB, green: apiary area EF, blue: apiary area CD). **B**: response permutation test plot, showing calculated (n=100) R2Y(cum) and Q2(cum) values vs. model values; y-axis intercepts: R2= (0.0, 0.357), Q2= (0.0, -0.636). **C**: V-shaped plot, displaying variable importance projection (VIP) scores (y-axis) and p(corr) values for the variables. Variables with VIP>1.2 and p(corr) >0.5 (marked in red) are considered as relevant for class separation and reliable.

**Figure S9:** OPLS-DA model for dataset 2 (all samples excl. QC samples), classified by apiary area [2+5+0 principal components; R2X (cum)= 0.589, R2(cum) =0.989, Q2(cum)=0.97]. **A**: OPLS-DA score scatter plot t[1]/to[1], colored according apiary area (red: apiary area AB, green: apiary area EF, blue: apiary area CD). **B**: response permutation test plot, showing calculated (n=100) R2Y(cum)and Q2(cum) values vs. model values; y-axis intercepts: R2= (0.0, 0.56), Q2= (0.0, -0.612). **C**: V-shaped plot, displaying variable importance projection (VIP) scores (y-axis) and p(corr) values for the variables. Variables with VIP>1.2 and p(corr) >0.5 (marked in red) are considered as relevant for class separation and reliable.

**Table S11:** Annotated honey constituents relevant for class separation between apiary areas, based on VIP and |p(corr)| values from OPLS-DA models of dataset 1 and 2 (Figure S8 and S9). ID level [1,2]. **1**, confident 2D structure, identified by reference standard match (retention time and MS/MS); **2**, tentative annotation by comparison of structural formula and MS/MS fragmentation pattern with data from databases or literature. **3**, no data available in literature or databases; compound tentatively assigned on the basis of the MS/MS fragmentation pattern (theoretical interpretation and/or comparison to related compounds) (annotation details, see Table S12). Mean peak areas in the respective apiary areas are colored with a threepartite color scale (red = low, white= medium, green= high abundance), boxplots illustrating the distribution of the metabolites in the apiary areas are depicted in Figure S10 and S11. Statistical comparison of peak areas between apiary areas was performed by Kruskal-Wallis One Way Analysis of Variance on Ranks* followed by pairwise multiple comparison with Dunn´s method** (p<0.05 marked in gray)

| **com-pound** | **RT [min]** | **neutral monoiso-topic mass** | **VIP value** | **\|p(corr)\|** | **mean peak area (AB)** | **mean peak area (CD)** | **mean peak area**  **(EF)** | **p-value (all apiary areas)*** | | **p-value (CD vs AB)**** | | **p-value (CD vs EF)**** | | **p-value (EF vs AB)**** | | **annotation^IDlevel^** | **compound class** | |  |
| --- | --- | --- | --- | --- | --- | --- | --- | --- | --- | --- | --- | --- | --- | --- | --- | --- | --- | --- | --- |
| **Dataset 1** | | | | | | | | | | | | | | | | | | | |
| **D1_d_1** | 3.04 | 286.1053 | 1.43 | 0.59 | 1.55E+07 | 1.47E+08 | 1.86E+07 | 0.002 | 0.002 | | 0.009 | | 1.000 | | salicin^1^ | | | phenyl glycoside | |
| **D1_d_2** | 3.55 | 130.063 | 1.84 | 0.62 | 8.41E+06 | 1.60E+08 | 3.31E+08 | <0.001 | 0.050 | | 1.000 | | <0.001 | | methyl oxopentanoic acid isomer 1^2^ | | | carboxylic acid | |
| **D1_d_3** | 3.92 | 176.0685 | 1.33 | 0.72 | 4.52E+07 | 4.68E+08 | 1.33E+08 | <0.001 | <0.001 | | 0.121 | | 0.043 | | isopropylmalic acid^2^ | | | dicarboxylic acid | |
| **D1_d_4** | 4.16 | 130.063 | 1.93 | 0.67 | 6.21E+06 | 2.28E+08 | 4.08E+08 | <0.001 | 0.026 | | 1 | | <0.001 | | methyl oxopentanoic acid isomer 2^2^ | | | carboxylic acid | |
| **D1_d_5** | 6.76 | 346.1628 | 1.38 | 0.65 | 5.10E+06 | 8.33E+07 | 3.73E+07 | 0.003 | 0.020 | | 1.000 | | 0.008 | | nepetaside or isomer 1^2^ | | | monoterpene glycoside | |
| **D1_d_6** | 7.08 | 346.1628 | 1.37 | 0.64 | 5.20E+06 | 9.28E+07 | 3.92E+07 | 0.002 | 0.016 | | 1.000 | | 0.004 | | nepetaside or isomer 2^2^ | | | monoterpene glycoside | |
| **D1_d_7** | 7.52 | 346.1628 | 1.41 | 0.64 | 6.33E+06 | 1.09E+08 | 4.91E+07 | 0.002 | 0.016 | | 1.000 | | 0.004 | | deoxycatalpol or isomer 1^2^ | | | monoterpene glycoside | |
| **D1_d_8** | 8.42 | 346.1628 | 1.23 | 0.56 | 1.02E+07 | 1.45E+08 | 4.20E+07 | 0.022 | 0.045 | | 1.000 | | 0.067 | | deoxycatalpol or isomer 2^2^ | | | monoterpene glycoside | |
| **D1_d_9** | 9.10 | 346.1628 | 1.26 | 0.60 | 1.33E+07 | 1.66E+08 | 5.74E+07 | 0.009 | 0.026 | | 1.000 | | 0.029 | | deoxycatalpol or isomer 3^2^ | | | monoterpene glycoside | |
| **D1_d_10** | 9.41 | 437.2315 | 1.29 | 0.71 | 1.42E+08 | 2.65E+07 | 2.55E+07 | 0.002 | 0.009 | | 1.000 | | 0.006 | | dicoumaroylspermidine isomer^2^ | | | hydroxycin-namoylamide | |
| **D1_d_11** | 10.59 | 686.2633 | 1.20 | 0.54 | 1.32E+08 | 1.93E+07 | 5.58E+07 | 0.011 | 0.011 | | 0.443 | | 0.143 | | deoxylamiol dihexoside^3^ | | | monoterpene glycoside | |
| **D1_d_12** | 17.35 | 272.0685 | 1.33 | 0.74 | 1.29E+08 | 1.89E+09 | 8.94E+08 | <0.001 | <0.001 | | 0.664 | | 0.005 | | pinobanksin or isomer^2^ | | | flavonoid | |
| **Dataset 2** | | | | | | | | | | | | | | | | | | | |
| **D2_d_1** | 0.98 | 123.032 | 1.29 | 0.53 | 5.57E+08 | 1.68E+09 | 1.02E+09 | 0.014 | 0.011 | | 0.295 | | 0.257 | | nicotinic acid^1^ | | | vitamin | |
| **D2_d_2** | 2.13 | 121.0891 | 1.51 | 0.67 | 1.31E+08 | 1.55E+09 | 1.15E+09 | <0.001 | 0.003 | | 1 | | 0.004 | | phenylethylamine or isomer^2^ | | | biogenic amine | |
| **D2_d_3** | 6.08 | 224.1412 | 1.27 | 0.50 | 2.60E+08 | 1.37E+09 | 3.51E+08 | 0.003 | 0.004 | | 0.006 | | 1 | | vomifoliol or isomer^2^ | | | megastigma-ne glycoside | |
| **D2_d_4** | 7.81 | 599.2631 | 1.41 | 0.73 | 7.55E+07 | 8.48E+08 | 1.87E+08 | <0.001 | <0.001 | | 0.118 | | 0.072 | | dicoumaroyl-caffeoylspermidine isomer 1^2^ | | | hydroxycin-namoylamide | |
| **D2_d_5** | 8.05 | 599.2631 | 1.24 | 0.59 | 5.41E+07 | 6.08E+08 | 1.35E+08 | 0.008 | 0.008 | | 0.419 | | 0.116 | | dicoumaroyl-caffeoylspermidine isomer 2^2^ | | | hydroxycin-namoylamide | |
| **D2_d_6** | 8.31 | 599.2631 | 1.23 | 0.51 | 8.37E+07 | 1.01E+09 | 1.65E+08 | 0.03 | 0.025 | | 0.157 | | 0.894 | | dicoumaroyl-caffeoylspermidine isomer 3^2^ | | | hydroxycin-namoylamide | |
| **D2_d_7** | 12.77 | 254.0579 | 1.51 | 0.71 | 7.44E+07 | 1.95E+09 | 7.64E+08 | <0.001 | 0.003 | | 1 | | 0.003 | | chrysin^1^ | | | flavonoid | |
| **D2_d_8** | 17.14 | 268.0736 | 1.56 | 0.71 | 2.31E+07 | 8.07E+08 | 2.51E+08 | <0.001 | 0.002 | | 1 | | 0.003 | | tectochrysin or isomer^2^ | | | flavonoid | |

**Table S12**: **Details for annotation of metabolites relevant for class separation between apiary areas.** Compound numbering as in Table S11. ID level [1,2]: **1**, confident 2D structure, identified by reference standard match (retention time and MS/MS); **2**, tentative annotation by comparison of structural formula and MS/MS fragmentation pattern with data from databases or literature. **3**, no data available in literature or databases; compound tentatively assigned on the basis of the MS/MS fragmentation pattern (theoretical interpretation and/or comparison to related compounds)

| **compound** | **RT [min]** | ***m/z* (experimental)** | **neutral monoisotopic mass** | **calculated neutral formula** | **Δ (ppm)** | | **MS/MS fragments** | | **ID^ID level^** | **Annotation source** | **Detected in bee product (example)** | **Compound class** |
| --- | --- | --- | --- | --- | --- | --- | --- | --- | --- | --- | --- | --- |
| **Dataset 1 (HESI negative mode ionization); Discriminant metabolites D1_d_1- D1_d_12** | | | | | | | | | | | | |
| **D1_d_1** | 3.04 | 331.1033 [M+HCOO]^-^ | 286.1053 | C_13_H_18_O_7_ | 2.84 | | 285.0982 (3) 123.0452 (100) 121.0195 (20) 93.0348 (5) | | salicin^1^ | reference compound | honey [17] | phenylglycoside |
| **D1_d_2** | 3.55 | 129.0556 | 130.063 | C_6_H_10_O_3_ | 7.51 | | 129.0558 (100) 101.0610 (5) 85.0660 (3) | | methyl oxopentanoic acid isomer 1^2^ | MonNA ML001451, ML001951 | Finnish dandelion honey [18] | carboxylic acid |
| **D1_d_3** | 3.92 | 175.0609 | 176.0685 | C_7_H_12_O_5_ | 4.8 | | 175.0609 (65) 157.0503 (10) 146.9611 (10) 131.0711 (10) 115.0399 (100) 113.0607 (40) 85.0658 (40) | | isopropylmalic acid^2^ | MzVault Bamba lab 598 polar metabolites stepped NCE |  | dicarboxylic acid |
| **D1_d_4** | 4.16 | 129.0556 | 130.063 | C_6_H_10_O_3_ | 7.51 | | 129.0558 (100) 101.0610 (5) 85.0660 (3) | | methyl oxopentanoic acid isomer 2^2^ | MonNA ML001451, ML001951 | Finnish dandelion honey [18] | carboxylic acid |
| **D1_d_5** | 6.76 | 391.1608 [M+HCOO]^-^ | 346.1628 | C_16_H_26_O_8_ | 0.97 | | 225.1128 (30) 197.1182 (20) 183.1024 (50) 113.0244 (100) 101.0246 (10) 95.0138 (20) 89.0245(25) 71.0138 (45) 59.0139 (55) | | nepetaside or isomer ^2^ | literature [19] |  | monoterpene glycoside |
| **D1_d_6** | 7.08 | 391.1608 [M+HCOO]^-^ | 346.1628 | C_16_H_26_O_8_ | 0.97 | | 225.1130 (45) 197.1180 (20) 183.1024 (75) 155.0714 (10) 139.1127 (15) 127.0763 (20) 113.0243 (85) 101.0243 (25) 89.0244 (35) 71.0138 (45) 59.0138 (100) | | nepetaside or isomer ^2^ | literature [19] |  | monoterpene glycoside |
| **D1_d_7** | 7.52 | 391.1612 [M+HCOO]^-^ | 346.1628 | C_16_H_26_O_8_ | 2.04 | | 225.1134 (30) 197.1183 (25) 183.1026 (80) 165.0921 (5) 161.0454 (10) 139.1129 (15) 119.0351 (20) 113.0244 (80) 101.0245 (30)89.0245 (35) 85.0296 (25) 71.0139 (50) 59.0139 (100) | | deoxycatalpol or isomer ^2^ | literature [20] |  | monoterpene glycoside |
| **D1_d_8** | 8.42 | 391.1613 [M+HCOO]^-^ | 346.1628 | C_16_H_26_O_8_ | 2.12 | | 285.1346 (5) 255.1238 (5) 225.1134 (80) 197.1184 (100) 183.1026 (95) 165.0921 (10) 155.0714 (40) 137.0609 (35) 127.0765 (75) 111.0816 (15) 93.0711 (15) 71.0139 (28) 59.0139 (40) | | deoxycatalpol or isomer ^2^ | literature [20] |  | monoterpene glycoside |
| **D1_d_9** | 9.10 | 381.1320 [M+Cl]^-^ | 346.1628 | C_16_H_26_O_8_ | 0.89 | | 225.11131 (40) 197.1182 (65) 183.1024 (100) 165.0916 (7) 155.0712 (20) 139.1182 (30) 137.0607 (27) 127.0763 (50) 101.0245 (10) 89.0244 (20) 71.0138 (50) 59.0138 (55) | | deoxycatalpol or isomer ^2^ | literature [20] |  | monoterpene glycoside |
| **D1_d_10** | 9.41 | 436.2239 | 437.2315 | C_25_H_31_O_4_N_3_ | 1.83 | | 436.2242 (10) 316.1665 (40) 273.1605 (5) 119.0501 (100) | | dicoumaroylspermidine isomer^2^ | literature [11] | rape bee pollen [11] | hydroxycinnamoylamide |
| **D1_d_11** | 10.59 | 685.2575 | 686.2633 | C_28_H_46_O_19_ | 3.28 | | 685.2567 (40) 523.2030 (15) 403.1610 (20) 361.1504 (25) 301.1292 (20) 199.0975 (100) 155.1077 (90) 137.0970 (25) 101.0244 (35) | | deoxylamiol dihexoside^3^ |  |  | monoterpene glycoside |
| **D1_d_12** | 17.35 | 271.061 | 272.0685 | C_15_H_12_O_5_ | 3.21 | | 271.0610 (100) 253.0505 (25) 225.0553 (5) 197.0608 (15) 161.0606 (10) 151.0035 (10) 125.0243 (15) 107.0137 (5) | | pinobanksin or isomer^2^ | literature [21] | honey [21] | flavonoid |
| **Dataset 2 (ESI positive mode ionization); Discriminant metabolites D2_d_1- D2_d_8** | | | | | | | | | | | | |
| **D2_d_1** | 0.98 | 124.0395 | 123.032 | C_6_H_5_O_2_N | | -2.77 | | 124.0395 (100) 96.0449 (5) 80.0502 (10) | nicotinic acid^1^ | reference compound | honey [12] | vitamin |
| **D2_d_2** | 2.13 | 122.0969 | 121.0891 | C_8_H_11_N | | 3.55 | | 122.0698 (3) 105.0704 (100) 95.0497 (3) 79.0550 (3) | phenylethylamine or isomer^2^ | MzCloud Ref 1191 | honey[22,23] | biogenic amine |
| **D2_d_3** | 6.08 | 225.1488 | 224.1412 | C_13_H_20_O_3_ | | 1.32 | | 225.1489 (15) 207.1384 (45) 151.0755 (10) 123.1172 (40) 85.0292 (100) | vomifoliol or isomer ^2^ | literature [24] | honey [25] | megastigmane glycoside |
| **D2_d_4** | 7.81 | 600.27 | 599.2631 | C_34_H_37_O_7_N_3_ | | -1.59 | | 454.2339 (90) 436.2231 (25) 308.1971 (20) 292.2020 (15) 275.1751 (15) 234.1124 (15) 204.1022 (85) 163.0392 (35) 147.0442 (100) | dicoumaroyl-caffeoylspermidine isomer 1^2^ | literature [14] | bee pollen [26] | hydroxycinnamoylamide |
| **D2_d_5** | 8.05 | 600.2701 | 599.2631 | C_34_H_37_O_7_N_3_ | | -1.39 | | 600.2694 (5) 454.2338 (100) 438.2389 (30) 308.1966 (15) 292.2017 (20) 275.1744 (10) 220.0970 (10) 204.1021 (95) 163.0391 (35) 147.0441 (100) | dicoumaroyl-caffeoylspermidine isomer 2^2^ | literature [14] | bee pollen [26] | hydroxycinnamoylamide |
| **D2_d_6** | 8.31 | 600.2703 | 599.2631 | C_34_H_37_O_7_N_3_ | | -1.09 | | 600.2683 (10) 454.2338 (80) 436.2233 (30) 308.1967 (10) 292.2023 (15) 275.1752 (10) 234.1129 (10) 204.1021 (95) 163.0390 (25) 147.0442 (100) | dicoumaroyl-caffeoylspermidine isomer 3^2^ | literature [14] | bee pollen [26] | hydroxycinnamoylamide |
| **D2_d_7** | 12.77 | 255.0655 | 254.0579 | C_15_H_10_O_4_ | | 1.39 | | 255.0652 (100) 153.0185 )3 | chrysin^1^ | reference compound | honey (frequent) [27] | flavonoid aglycone |
| **D2_d_8** | 17.14 | 269.0811 | 268.0736 | C_16_H_12_O_4_ | | 0.95 | | 269.0809 (100) 254.0580 (5) 226.0622 (10) | tectochrysin or isomer^2^ | MoNA BML00756 | honey (frequent) [3] | flavonoid aglycone |

**Figure S10:** Boxplots showing peak areas of annotated constituents relevant for class separation between apiary areas in OPLS-DA model of dataset 1 (see Table S11 and S12). Solid line: median, dashed line: average.

**Figure S11:** Boxplots showing peak areas of annotated constituents relevant for class separation between apiary areas in OPLS-DA model of dataset 2 (see Table S11 and S12). Solid line: median, dashed line: average.

References

1. Sumner, L. W. *et al.* Proposed minimum reporting standards for chemical analysis Chemical Analysis Working Group (CAWG) Metabolomics Standards Initiative (MSI). *Metabolomics : Official journal of the Metabolomic Society* **3,** 211–221 (2007).

2. Blaženović, I., Kind, T., Ji, J. & Fiehn, O. Software Tools and Approaches for Compound Identification of LC-MS/MS Data in Metabolomics. *Metabolites* **8** (2018).

3. Koulis, G. A. *et al.* Thorough Investigation of the Phenolic Profile of Reputable Greek Honey Varieties: Varietal Discrimination and Floral Markers Identification Using Liquid Chromatography-High-Resolution Mass Spectrometry. *Molecules (Basel, Switzerland)* **27** (2022).

4. Alipieva, K., Kokubun, T., Taskova, R., Evstatieva, L. & Handjieva, N. LC–ESI-MS analysis of iridoid glucosides in Lamium species. *Biochemical Systematics and Ecology* **35,** 17–22 (2007).

5. Kokotou, M. G., Mantzourani, C., Babaiti, R. & Kokotos, G. Study of the Royal Jelly Free Fatty Acids by Liquid Chromatography-High Resolution Mass Spectrometry (LC-HRMS). *Metabolites* **10** (2020).

6. Steimer, S. S., Kourtchev, I. & Kalberer, M. Mass Spectrometry Characterization of Peroxycarboxylic Acids as Proxies for Reactive Oxygen Species and Highly Oxygenated Molecules in Atmospheric Aerosols. *Analytical chemistry* **89,** 2873–2879 (2017).

7. Montoro, P., D'Urso, G., Kowalczyk, A. & Tuberoso, C. I. G. LC-ESI/LTQ-Orbitrap-MS Based Metabolomics in Evaluation of Bitter Taste of Arbutus unedo Honey. *Molecules (Basel, Switzerland)* **26** (2021).

8. Isidorov, V. A., Czyżewska, U., Jankowska, E. & Bakier, S. Determination of royal jelly acids in honey. *Food Chemistry* **124,** 387–391 (2011).

9. Tomás-Barberán, F. A., Martos, I., Ferreres, F., Radovic, B. S. & Anklam, E. HPLC flavonoid profiles as markers for the botanical origin of European unifloral honeys. *J. Sci. Food Agric.* **81,** 485–496 (2001).

10. Wang, Q., Cai, W.-J., Yu, L., Ding, J. & Feng, Y.-Q. Comprehensive Profiling of Phytohormones in Honey by Sequential Liquid-Liquid Extraction Coupled with Liquid Chromatography-Mass Spectrometry. *Journal of agricultural and food chemistry* **65,** 575–585 (2017).

11. Zhang, H., Liu, R. & Lu, Q. Separation and Characterization of Phenolamines and Flavonoids from Rape Bee Pollen, and Comparison of Their Antioxidant Activities and Protective Effects Against Oxidative Stress. *Molecules (Basel, Switzerland)* **25** (2020).

12. El-Hawiet, A., Elessawy, F. M., El Demellawy, M. A. & El-Yazbi, A. F. Green fast and simple UPLC-ESI-MRM/MS method for determination of trace water-soluble vitamins in honey: Greenness assessment using GAPI and analytical eco-scale. *Microchemical Journal* **181,** 107625 (2022).

13. Jerković, I., Hegić, G., Marijanović, Z. & Bubalo, D. Organic extractives from Mentha spp. honey and the bee-stomach: methyl syringate, vomifoliol, terpenediol I, hotrienol and other compounds. *Molecules (Basel, Switzerland)* **15,** 2911–2924 (2010).

14. Li, Z. *et al.* Deep Annotation of Hydroxycinnamic Acid Amides in Plants Based on Ultra-High-Performance Liquid Chromatography-High-Resolution Mass Spectrometry and Its In Silico Database. *Analytical chemistry* **90,** 14321–14330 (2018).

15. Tugizimana, F., Steenkamp, P. A., Piater, L. A. & Dubery, I. A. A Conversation on Data Mining Strategies in LC-MS Untargeted Metabolomics: Pre-Processing and Pre-Treatment Steps. *Metabolites* **6** (2016).

16. Tugizimana, F., Steenkamp, P. A., Piater, L. A., Labuschagne, N. & Dubery, I. A. Unravelling the Metabolic Reconfiguration of the Post-Challenge Primed State in Sorghum bicolor Responding to Colletotrichum sublineolum Infection. *Metabolites* **9** (2019).

17. McLoone, P. *et al.* Qualitative phytochemical analysis and in vitro investigation of the immunomodulatory properties of honeys produced in Kazakhstan. *Natural product research* **37,** 996–1001 (2023).

18. Kortesniemi, M. *et al.* NMR profiling clarifies the characterization of Finnish honeys of different botanical origins. *Food Research International* **86,** 83–92 (2016).

19. Zou, L., Li, X., Shi, Q. & Feng, F. An effective integrated method for comprehensive identification of eighty-five compounds in Zhi-Zi-Da-Huang decoction by HPLC-DAD-ESI-MS (TOF) and HPLC-DAD-ESI-MS/MS (QqQ) without the help of reference standards. *Anal. Methods* **6,** 4312–4327 (2014).

20. Lei, H. *et al.* Comprehensive profiling of the chemical components and potential markers in raw and processed Cistanche tubulosa by combining ultra-high-performance liquid chromatography coupled with tandem mass spectrometry and MS/MS-based molecular networking. *Analytical and bioanalytical chemistry* **413,** 129–139 (2021).

21. Koulis, G. A. *et al.* Honey Phenolic Compound Profiling and Authenticity Assessment Using HRMS Targeted and Untargeted Metabolomics. *Molecules (Basel, Switzerland)* **26** (2021).

22. Pereira, V., Pontes, M., Câmara, J. S. & Marques, J. C. Simultaneous analysis of free amino acids and biogenic amines in honey and wine samples using in loop orthophthalaldeyde derivatization procedure. *Journal of chromatography. A* **1189,** 435–443 (2008).

23. Yu, W. *et al.* Chemical composition and anti-inflammatory activities of Castanopsis honey. *Food & function* **14,** 250–261 (2023).

24. Zhao, Z.-H. *et al.* Leonurus japonicus Houtt. (Motherwort): Systematic research through chemical profiling, stability under controlled conditions and pharmacokinetic analysis on screening Q-markers for quality control. *Journal of pharmaceutical and biomedical analysis* **213,** 114707 (2022).

25. Jerković, I., Kuś, P. M., Tuberoso, C. I. G. & Šarolić, M. Phytochemical and physical-chemical analysis of Polish willow (Salix spp.) honey: identification of the marker compounds. *Food Chemistry* **145,** 8–14 (2014).

26. Zhang, X., Yu, M., Zhu, X., Liu, R. & Lu, Q. Metabolomics reveals that phenolamides are the main chemical components contributing to the anti-tyrosinase activity of bee pollen. *Food Chemistry* **389,** 133071 (2022).

27. Preti, R. & Tarola, A. M. Chemometric evaluation of the antioxidant properties and phenolic compounds in Italian honeys as markers of floral origin. *Eur Food Res Technol* **248,** 991–1002 (2022).
